# Supplementary material for: Emulator-based Bayesian optimization for efficient multi-objective calibration of an individual-based model of malaria
Source: Nat Commun. 2021 Dec 10;12:7212. doi: 10.1038/s41467-021-27486-z (PMC8664949; doi:10.1038/s41467-021-27486-z)
Supplement: Supplementary file 1 — Supplementary Information [file 41467_2021_27486_MOESM1_ESM.pdf]

Supplementary Information for

Emulator-based Bayesian optimization for efficient multi-objective calibration of an individual-based model of malaria

Authors: Theresa Reiker[1,2], Monica Golumbeanu[1,2], Andrew Shattock[1,2], Lydia Burgert[1,2], Thomas A. Smith[1,2], Sarah Filippi[3], Ewan Cameron[4,5,6], Melissa A. Penny[1,2*].

Affiliations:

[1]Swiss Tropical and Public Health Institute, Basel, Switzerland.

[2]University of Basel, Basel, Switzerland.

[3] Imperial College London, UK.

[4] Malaria Atlas Project, Big Data Institute, University of Oxford, Oxford, UK.

[5] Curtin University, Perth, Australia.

[6] Telethon Kids Institute, Perth Children's Hospital, Perth, Australia.

*Correspondence to: melissa.penny@unibas.ch

This file includes:

Supplementary Notes 1 and 2

Supplementary Figures 1 to 32

Supplementary Tables 1 to 6

Contents

1 Supplementary Note 1: Malaria Transmission Model 3
1.1 Main features 3
1.2 Infection of the human host 4
1.2.1 Differential feeding by mosquitoes depending on body surface area 4
1.2.2 Control of pre-erythrocytic stages 4
1.2.3 Course of infection in the human host 5
1.2.4 Infectivity of the human host 6
1.3 Morbidity 7
1.3.1 Acute morbidity (uncomplicated clinical cases) 7
1.3.2 Severe disease 8
1.3.3 Mortality 8
2 Supplementary Note 2: Calibration Approach and Data Summary 3
2.1 Objectives: Epidemiological data and loss functions 5
2.1.1 Age pattern of incidence after intervention 5
2.1.2 Age patterns of prevalence 5
2.1.3 Age patterns of parasite density 6
2.1.4 Age pattern of number of concurrent infections 7
2.1.5 Age pattern of incidence of clinical malaria 8
2.1.6 Age pattern of incidence of clinical malaria: infants 9
2.1.7 Age pattern of threshold parasite density for clinical attacks 9
2.1.8 Hospitalization rate in relation to prevalence in children 10
2.1.9 Age pattern of hospitalization: severe malaria 12
2.1.10 Malaria specific mortality in children (< 5 years old) 13
2.1.11 Indirect malaria infant mortality rate 14
2.2 Supplementary Table 3 15
3 Emulator performance 21
4 Adaptive sampling: selected points 25
4.1 GP-BO 25
4.2 GPSG-BO 26
5 OpenMalaria: Final simulator fit 27
6 Validation 33
7 OpenMalaria simulated epidemiology 35
8 Log prior distributions 41
9 Ranger importance 42
10 References 42

1 SUPPLEMENTARY NOTE 1: MALARIA TRANSMISSION MODEL

1.1 Main features

We test our calibration algorithm on OpenMalaria, an individual-based model of malaria dynamics. To provide context of the model's structure and the role of the fitted parameters (see Supplementary Note 1), we here briefly describe its main features and key equations. This description is adapted from that provided in Smith et al. 2012 [1] and Smith et al. 2006 [2]. Full details of all model components can be found in *The American Journal of Tropical Medicine and Hygiene*, Volume 75, Issue 2 Supplement (2006).

OpenMalaria features discrete individual-based stochastic simulations of malaria in humans in 5-day time steps. Every infection and individual are characterized by a set of continuous state variables, namely, parasite densities, infection durations, and immune status. Key processes and relationships regarding the course of infection simulated by model include the attenuation of inoculations, acquired pre-erythrocytic immunity, acquired blood-stage immunity, morbidity (acute and severe) and mortality (malaria-specific and indirect), anemia, and the infection of vectors as a function of parasite densities in the human. Other model components include a vector model and a case management system. All individual components have previously been well documented [1,2]. A visual summary of the model with references to further details on each component is provided in Supplementary Figure 1.

In our current recalibration only the original (base) model variant is used to test our new approach [1]. Parameters estimated during the calibration process are highlighted and summarized in Supplementary Table 1 at the end of this section. Other parameter values were drawn from the literature or were calibrated to separate data: for example, the empirical parasite density model of Maire et al. 2006 [3] was calibrated to malaria therapy data [4] and not recalibrated at the population level.

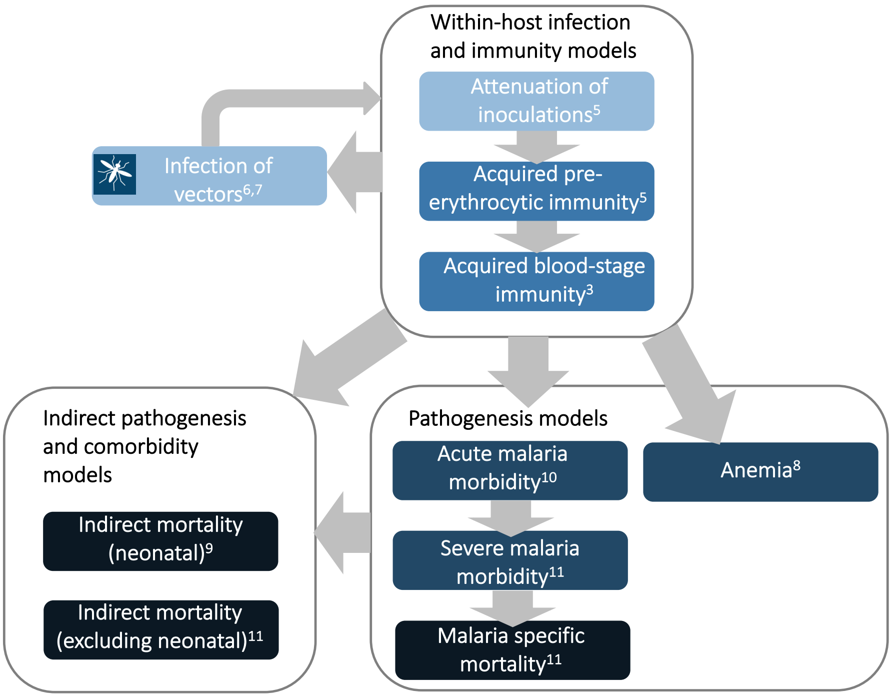

Supplementary Figure 1. Visual summary of OpenMalaria model components with references to original publications. References from top to bottom and left to right: Attenuation of inoculations[5], Infection of vectors[6,7], Acquired pre-erythrocytic immunity[5], Anemia[8], Acquired blood-stage immunity[3], Acute malaria morbidity[10], Indirect mortality (neonatal)[9], Severe malaria morbidity[11], Indirect mortality (excluding neonatal)[11], Malaria specific mortality[11].

1.2 Infection of the human host

The seasonal pattern of entomological inoculation rate (EIR) determines seasonal pattern of transmission and thus the parasite densities in the individual, modified by natural or acquired immunity and interventions [2].

1.2.1 Differential feeding by mosquitoes depending on body surface area

In the base model, the expected number of entomological inoculations experienced by individual i of age a at time t is

$$E_a(i,t) = \frac{E_{max}(t)A(a(i,t))}{A_{max}} \tag{1}$$

where $E_{max}(t)$ refers to the annual entomological inoculation rate (EIR) computed from human bait collections on adults and $A()$, is the individual's availability to mosquitoes, assumed to be proportional to average body surface area, depending only on age $a(i,t)$. $A\big(a(i,t)\big)$ increases with age up to age 20 years where it reaches a value of A_{max} (the average body surface of people ≥ 20 years old in the same population).

The biting rate in relation to human weight is based on data from The Gambia published by Port and others [12], where the proportion of mosquitoes that had fed on a host were analyzed in relation to the host's contribution to the total biomass and surface area of people sleeping in one mosquito net [5].

1.2.2 Control of pre-erythrocytic stages

The number of infective bites received per unit time for each individual i, adjusted by age, is given by Eq. 1 above. A survival function $S(i,t)$ defines the probability that the progeny of an inoculation survives to give rise to a patent blood stage infection, i.e., the proportion of inoculations that result in infections or the susceptibility of individual i at time t. The force of infection is modelled as

$$\lambda(i,t) = S(i,t)E_a(i,t), \tag{2}$$

where $E_a(i,t)$ is the expected number of entomological inoculations endured by individual i at time t, adjusted for age and individual factors, and the number of infections $h(i,t)$ acquired by individual i in five-day time step t, follows a Poisson distribution:

$$h(i,t) \sim Poisson\big(\lambda(i,t)\big). \tag{3}$$

The susceptibility of individual i at time t, $S(i,t)$ is defined as:

$$S(i,t) = \left(S_\infty + \frac{1-S_\infty}{1+\frac{E_a(i,t)}{E^*}}\right)\left(S_{imm} + \frac{1-S_{imm}}{1+\left(\frac{X_p(i,t)}{X_p^*}\right)^{\gamma_p}}\right), \tag{4}$$

where $S_{imm}, X_p^*, E^*, \gamma_p$ and S_∞ are constants representing the lower limit of success probability of inoculations in immune individuals, critical value of cumulative number of entomologic inoculations, critical value of $E_a(i,t)$, steepness of relationship between success of inoculation and $X_p(i,t)$, and, the lower limit of success probability of inoculations at high where $E_a(i,t)$, respectively. Here

$$X_p(i,t) = \int_{t-a(i,t)}^{t} E_a(i,\tau)d\tau \tag{5}$$

S_∞ and E^* are fixed to $S_\infty = 0.049$, and $E^* = 0.032$ inoculations/person-night and are detailed in [5].

1.2.3 Course of infection in the human host

The model for each individual infection j in host i comprises a time series of parasite densities. The base model for infection within humans is described in Maire et al. 2006 [3]. In brief, the duration of each infection, τ_{max} is sampled from

$$\ln(\tau_{max}(i,j)) \sim Normal(5.13,0.80), \tag{6}$$

parameterised against malaria therapy data [4] and detailed in Maire et al. 2006 [3]. In the absence of previous exposure or concurrent infections, the log density of infection j in host i at each time point, $\tau = 0,1, \dots, \tau_{max}(i,j)$ is normally distributed with expectation

$$\ln(y_0(i,j,\tau)) = \ln d(i) + \ln y_G(\tau, \tau_{\max}), \tag{7}$$

where $y_G(\tau, \tau_{max})$ is taken from a statistical description of parasite densities in malariatherapy patients and $d(i)$ describes between-host variation with a log-normal distribution with variance σ_i^2.

We consider the possibility of multiple concurrent infections in the same individual at the same time. Exposure to asexual blood stages is measured by

$$X_y(i,j,t) = \int_{t-a}^{t} Y(i,\tau)\, d\tau - \int_{t_{0,j}}^{t} y(i,j,\tau) d\tau, \tag{8}$$

where $Y(i,\tau)$ is the total parasite density of individual i at time τ and $y(i,j,\tau)$ is the density of infection j in individual i at time τ and

$$X_h(i,t) = \int_{t-a}^{t} h(i,\tau)\, d\tau - 1. \tag{9}$$

In the presence of previous exposure and co-infection, the expected log density for each concurrent infection is then:

$$E(\ln(y(i,j,\tau))) = D_y(i,t) D_h(i,t) D_m(i,t) \ln(y_0(i,j,\tau)) + \ln\left(\frac{D}{M(i,t)} + 1 - D_x\right), \tag{10}$$

where $M(i,t)$ is the total multiplicity of infection of in individual i at time t, and

$$D_y(i,t) = \frac{1}{1 + \frac{X_y(i,j,t)}{X_y^*}}, \tag{11}$$

where $X_y(i,j,t) = \sum_{t-a}^{t} Y(i,t) - \sum_{t_{0,j}}^{t} y(i,j,\tau)$ (note that a continuous time approximation to this is given in the original publications [3,5] and hence measures the cumulative parasite load. Furthermore

$$D_h(i,t) = \frac{1}{1 + \frac{X_h(i,t)}{X_h^*}}, \tag{12}$$

where, $X_h(i,t) = \sum_{t-a}^{t} h(i,\tau) - 1$, the number of inoculations since birth, excluding the one under consideration, which measures the diversity of inocula experienced by the host up to the time point under consideration.

$$D_m(i,t) = 1 - \alpha_m \exp\left(-\frac{0.693a(i,t)}{a_m^*}\right) \quad (13)$$

which measures the effect of maternal immunity. X_y^*, X_h^*, D_x, a_m^*, and α_m are all constants estimated in the fitting process. These constants are described in Supplementary Table 1, or further in Maire et al. 2006 [3].

Variation within individuals described as $\sigma_y^2(i,j,\tau)$, where

$$\sigma_y^2(i,j,\tau) = \frac{\sigma_0^2}{1 + \frac{X_h(i,t)}{X_v^*}}, \quad (14)$$

and σ_o^2 and X_v^* are constants, described in Supplementary Table 1.

The simulated density of infection j in individual i at time τ is then drawn from a normal distribution:

$$\ln\big(y(i,j,\tau)\big) \sim Normal\ \Big(E\big(\ln\big(y(i,j,\tau)\big)\big), \sigma_y^2(i,j,\tau)\Big). \quad (15)$$

The total density of all infections in individual i at time t is then the sum of the densities of concurrent infections j

$$Y(i,t) = \sum_j y\big(i,j,\tau(i,j)\big). \quad (16)$$

1.2.4 Infectivity of the human host

The model infectivity of the human host is described in Ross 2006 where infectivity of individual I at time t is given by the distributed lag model:

$$\Upsilon(i,t) = \beta_1 Y(i,t-2) + \beta_2 Y(i,t-3) + \beta_3 Y(i,t-4), \quad (17)$$

where t is in 5-day units and

$$\ln\Big(y_g(i,t)\Big) \sim Normal\big(\ln\big(\rho\Upsilon(i,t)\big), \sigma_g^2\big), \quad (18)$$

where $\beta_1, \beta_2, \beta_3, \rho, \sigma_g^2$ are constants representing contributions of past infections to gametocyte densities (detailed in Supplementary Table 1), and to be calibrated at the population level. We define

$$\Pr\big(y_g(i,t) > y_g^*\big) = \Phi\left[\frac{\ln\big(\rho\Upsilon(i,t)\big) - \ln(y_g^*)}{\sigma_g}\right] = \Phi\left[\frac{\ln\big(\Upsilon(i,t)\big)}{\sigma_g} + \rho^*\right], \quad (19)$$

where Φ is the cumulative normal distribution, y_g^* is the density of female gametocytes necessary for infection of the mosquito, and $\rho^* = \frac{\ln(\rho) - \ln(y_g^*)}{\sigma_g}$ is constant (depending on the blood meal volume, gametocyte viability and system variability). Thus, the proportion of mosquitoes infected by individual i at time t is defined as

$$I_m(i,t) = \big[\Pr\big(y_g(i,t) > y_g^*\big)\big]^2, \quad (20)$$

and the probability of a mosquito becoming infected during any feed is

$$\kappa_u(t) = \eta \frac{\sum_i A\big(a(i,t)\big) I_m(i,t)}{\sum_i A\big(a(i,t)\big)} \quad (21)$$

where η is a constant scale factor and to be calibrated.

We define $\kappa_u^{(0)}(t)$ as the value of $\kappa_u(t)$ in the simulation of an equilibrium scenario to which an intervention has been applied. Let $E_{max}^{(0)}(t+l_v)$ be the corresponding entomologic inoculation rate. $\kappa_u^{(1)}(t)$ and $E_{max}^{(1)}(t+l_v)$ are the corresponding values for the intervention scenario. Then

$$E_{max}^{(1)}(t+l_v) = \frac{E_{max}^{(0)}(t+l_v)\kappa_u^{(1)}(t)}{\kappa_u^{(0)}(t)}, \tag{22}$$

where l_v corresponds to the duration of the sporogenic cycle in the vector, which we approximate with two time steps (10 days). $\frac{E_{max}^{(0)}(t+l_v)\kappa_u^{(1)}(1)}{\kappa_u^{(0)}(t)}$ is the total vectorial capacity)

1.3 Morbidity

In order to simulate the clinical state of individual i at time t, for each five-day time step 5 independent samples from the simulated parasite density distribution are drawn for each concurrent infection j.

1.3.1 Acute morbidity (uncomplicated clinical cases)

The model for an episode of acute morbidity was originally described in [10] and occurs in individual i at time t with probability

$$P_m(i,t) = \frac{Y_{max}(i,t)}{Y^*(i,t) + Y_{max}(i,t)}, \tag{23}$$

where $Y^*$is the pyrogenic threshold and Y_{max} is the maximum density of five daily densities sampled during the five-day interval t.

The pyrogenic threshold changes over time following

$$\frac{dY^*(i,t)}{dt} = f_1\big(Y(i,t)\big)f_2\big(Y^*(i,t)\big) - \varpi Y^*(i,y), \tag{24}$$

where $f_1\big(Y(i,t)\big)$ is a function describing the relationship between accrual of tolerance and the parasite density $Y(i,y)$; $f_2(Y^*(i,t))$ describes the saturation of this accrual process at high values of Y^* and $\varpi Y^*(i,t)$ determines the decay threshold with first-order kinetics, ensuring that the parasite tolerance is short-lived [10].

Here $f_1\big(Y(i,t)\big)$ is defined to ensure that the stimulus is not directly proportional to Y but rather that it asymptotically reaches a maximum at high values of Y:

$$f_1\big(Y(i,t)\big) = \frac{\alpha Y(i,t)}{Y_1^* + Y(i,t)}. \tag{25}$$

At high values of Y^*, a higher parasite load is required to achieve the same increase:

$$f_2\big(Y^*(i,t)\big) = \frac{1}{Y_2^* + Y^*(i,t)}. \tag{26}$$

Thus, the pyrogenic threshold $Y^*$is defined to follow

$$\frac{dY^*(i,t)}{dt} = \frac{\alpha Y(i,t)}{(Y_1^* + Y(i,t))(Y_2^* + Y^*(i,t))} - \varpi Y^*(i,t), \tag{27}$$

and the initial condition $Y^*(i,0) = Y_0^*$ at the birth of the host, where α, ϖ Y_0, Y_1^* and Y_2^* are targets of the calibration, and are defined in Supplementary Table 1.

1.3.2 Severe disease

The model for severe disease was described in Ross et al. 2006 [11] and two different classes of severe episodes are considered by the model, $B_1$and B_2. $P_{B1}(i,t)$ is the probability that an acute episode (A) is of class B_1 and

$$P_{B_1}(i,t) = \Pr(H(i,t) \in B_1 | H(i,t) \in A) = \frac{Y_{max}(i,t)}{Y_{B1}^{*} + Y_{max}(i,t)}, \tag{28}$$

where Y_{B1}^{*} is a constant to be calibrated and $H(i,t)$ is the clinical status of individual i at time t.

Class B_2 of severe malaria episodes occurs when an otherwise uncomplicated episode coincides with some other insult, which occurs with risk

$$F\big(a(i,t)\big) = \frac{F_0}{1 + \left(\frac{a(i,t)}{a_F^{*}}\right)}, \tag{29}$$

where F_0 is the limiting value of $F\big(a(i,t)\big)$ at birth and a_F^{*} is the age at which it is halved, and both are to be calibrated.

The probability that individual i experiences an episode belonging to class B_2 at time t, conditional on there being a clinical episode at that time is

$$P_{B_2}(i,t) = \Pr(H(i,t) \in B_2 \mid H(i,t) \in A) = F(a(i,t)). \tag{30}$$

The age ant time specific risk of severe malaria morbidity conditional on a clinical episode is then given by

$$P_B(i,t) = P_{B_1}(i,t) + P_{B_3}(i,t) - P_{B_1}(i,t)P_{B_2}(i,t). \tag{31}$$

1.3.3 Mortality

Malaria deaths in hospital are a random sample of admitted severe malaria cases, with age-dependent sampling fraction $Q_h(a)$, the hospital case fatality rate, derived from the data of Reyburn et al. (2004) [13]. The original model was described in Ross et al. 2006 [11].

The severe malaria case fatality in the community for age group a, $Q_c(a)$ is estimated as

$$Q_c(a) = \frac{Q_h(a)\phi_1}{1 - Q_h(a) + Q_h(a)\phi_1}, \tag{32}$$

where ϕ_1 the estimated odds ratio for death in the community compared to death in in-patients is an age-independent constant to be calibrated and $Q_h(a)$ is the hospital case fatality rate. The total malaria mortality is the sum of the hospital and community malaria deaths.

The risk of neonatal mortality attributable to malaria (death in class D_1) in first pregnancies is set equal to $0.3\mu_{PG}$ where

$$\mu_{PG} = \mu_{max}\left[1 - \exp\left(-\frac{x_{PG}}{x_{PG}^{*}}\right)\right], \tag{33}$$

where x_{PG} is related to x_{MG}, the prevalence in simulated individuals 20-24 ears of age via

$$x_{PG} = 1 - \frac{1}{1 + \left(\frac{x_{MG}}{x_{MG}^{*}}\right)} \tag{34}$$

and x_{MG}^{*} and x_{PG}^{*} are constants to be calibrated and are detailed in Supplementary Table 1.

An indirect death in class D_2 is provoked at time t, conditional on there being a clinical episode at that time with probability $P_{D2}(i,t)$ where

$$P_{D_2}(i,t) = \Pr(H(i,t) \in D_2 | H(i,t) \in A), \tag{35}$$

and

$$P_{D_2}(i,t) = \frac{Q_D}{1 + \left(\frac{a(i,t)}{a_F^*}\right)}, \tag{36}$$

where Q_D is limiting value of $P_{D_2}(i,t)$ at birth and a_F^* is a constant to be calibrated. Deaths in class D_2 occur 30 days (six time steps) after the provoking episode.

No.[*]	θ^+	Parameter	Meaning	Unit/ dimension	Prior	GA-O estimate (Smith et al. 2012, model R0001)[1]	New estimate GP-BO (Reiker et al.2021)	New estimate GPSG-BO (Reiker et al.2021)
1	--	$-\ln(1-S_\infty)$	S_∞ = Lower limit of success probability of inoculations at high $E_a(i,t)$	Proportion	--	0.051	0.051	0.051
2	--	E^*	Critical value of $E_a(i,t)$	Inoculations/ person-night	--	0.032	0.032	0.032
3	1[a]	S_{imm}	Lower limit of success probability of inoculations in immune individuals	Proportion	$\exp(N(\log(0.14),2))$	0.138	0. 196	0.036
4	3	X_p^*	Critical value of cumulative number of entomologic inoculations	Inoculations	$\exp(N(\log(1514),2))$	1,514.4	1,954.8	4,972.2
5	2	γ_p	Steepness of relationship between success of inoculation and $X_p(i,t)$	Dimensionless constant	$\exp(N(\log(1),1))$	2.037	1.291	1.871
6	23	σ_i^2	Variation between hosts on parasite densities (variance of log-normal distribution)		$\exp(N(\log(10.17),0.6))$	10.174	11.729	9.689
7	5	X_y^*	Critical value of cumulative number of parasite days	Parasite-days/μL x 10^{-7}	$\exp(N(\log(3.52x10^7),2))$	3.516	593.661	1.216
8	4	X_h^*	Critical value of cumulative number of infections	Infections	$\exp(N(\log(97.3),2))$	97.335	54.082	89.759
9	7	$\ln(1-\alpha_m)$	α_m = Maternal protection at birth	Dimensionless	$-\log(1-Beta(8,2))$	2.330	1.770	1.266
10	8	α_m^*	Decay of maternal protection	Per year	$\exp(N(\log(1.8),0.5))$	2.531	1.279	1.551
11	9	σ_0^2	Fixed variance component for densities	$[\ln(\text{density})]^2$	$\exp(N(\log(0.66),2))$	0.656	5.838	1.440
12	6	X_v^*	Critical value of cumulative number of infections for variance in parasite densities	Infections	$\exp(N(\log(5),1))$	0.916	3.959	7.226
13	14	Y_2^*	Critical value of $Y^*(i,t)$ in determining increase in Y^*	Parasites/μL	$\exp(N(\log(5000),1))$	6,502.26	6,560.08	13,485.57
14	10	α	Factor determining increase in $Y^*(i,t)$	$\text{Parasites}^2\mu L^{-2}\text{day}^{-}$	$\exp(N(\log(142602),1))$	142,602	63,220.5	119,502

15	22	$\boldsymbol{\nu_1}$	Density bias (non Garki)	Dimensionless	$\mathbf{exp}\left(\boldsymbol{N}(\mathbf{log}(\mathbf{0.177}), \mathbf{0.6})\right)$	0.177	0.123	0.159
16	--	$\boldsymbol{\sigma_2}$	Mass action parameter	Dimensionless		1	1	1
17	18	$\mathbf{log}\,\boldsymbol{\phi_1}$	Case fatality for severe episodes in the community compared to hospital	Log odds	$\mathbf{exp}\left(\boldsymbol{N}(\mathbf{log}(\mathbf{2.09}), \mathbf{0.3})\right)$	0.736	0.340	0.285
18	20[b]	$\boldsymbol{Q_D}$	Co-morbidity intercept relevant to indirect mortality	Proportion	$\mathbf{exp}\left(\boldsymbol{N}(\mathbf{log}(\mathbf{0.019}), \mathbf{1})\right)$	0.019	0.019	0.023
19	19[c]	$\boldsymbol{Q_n}$	Non-malaria intercept for infant mortality	Deaths / 1000 live births	$\mathbf{exp}\left(\boldsymbol{N}(\mathbf{log}(\mathbf{49.5}), \mathbf{1})\right)$	49.539	46.5095	40.163
20	21	$\boldsymbol{\nu_0}$	Density bias (Garki)	Dimensionless	$\mathbf{exp}\left(\boldsymbol{N}(\mathbf{log}(\mathbf{4.79}), \mathbf{0.2})\right)$	4.796	3.739	5.618
21	15	$\boldsymbol{Y^*_{\mathbf{B_1}}}$	Parasitaemia threshold for severe episodes type $\mathbf{B_1}$	Parasites/$\boldsymbol{\mu}$L	$\mathbf{exp}\left(\boldsymbol{N}(\mathbf{log}(\mathbf{250000}), \mathbf{0.8})\right)$	784,456	849,046	484,122
22	--	--	Immune penalty		--	1	1	1
23	--	--	Immune effector decay		--	0	0	0
24	16[d]	$\boldsymbol{F_0}$	Prevalence of co-morbidity/susceptibility at birth relevant to severe episodes ($\boldsymbol{B_2}$)	proportion	$\mathbf{exp}\left(\boldsymbol{N}(\mathbf{log}(\mathbf{0.092}), \mathbf{0.5})\right)$	0.097	0.078	0.094
25	11	$\dfrac{\mathbf{log}\,\mathbf{2}}{\boldsymbol{\bar{\omega}}}$	$\boldsymbol{Y^*}$(pyrogenic threshold) half-life	Years	$\mathbf{log}(\mathbf{2}) / \mathbf{exp}\left(\boldsymbol{N}(\mathbf{log}(\mathbf{2.52}), \mathbf{1})\right)$	0.275	0.468	0.516
26	13	$\boldsymbol{Y^*_1}$	Critical value of parasite density in determining increase in $\boldsymbol{Y^*}$	Parasites/$\boldsymbol{\mu}$L	$\mathbf{exp}\left(\boldsymbol{N}(\mathbf{log}(\mathbf{6}), \mathbf{2})\right)$	0. 597	1.665	0.477
27	--	--	Asexual immunity decay		--	0	0	0
28	12	$\boldsymbol{Y^*_0}$	Pyrogenic threshold at birth	Parasites/$\boldsymbol{\mu}$L	$\mathbf{exp}\left(\boldsymbol{N}(\mathbf{log}(\mathbf{296.3}), \mathbf{1})\right)$	296.302	90.938	201.671
29	--	--	Idete multiplier	Dimensionless	--	2.798	2.799	2.799
30	17	$\boldsymbol{a^*_F}$	Critical age for co-morbidity	Years	$\mathbf{exp}\left(\boldsymbol{N}(\mathbf{log}(\mathbf{0.225}), \mathbf{0.8})\right)$	0.117	0.138	0.087

Supplementary Table 1: Names and details of OpenMalaria core parameters. GA-O = Genetic algorithm optimization, GP-BO = Gaussian process-based Bayesian optimization, GPSG-BO = Gaussian process stacked generalization-based Bayesian optimization.

[*] Parameter number assigned for simulations in OpenMalaria scenarios, some parameters here are used in model variants and not in the base model. Listed for completeness; [+]Parameter number $\boldsymbol{\theta_i}$ assigned for the optimisation problem. $\boldsymbol{\theta}$ is drawn from the unit cube and determines the quantiles of the prior for the parameter value. [a] quantile = $\boldsymbol{\theta} * \mathbf{0.8372102}$. [b] quantile = $\boldsymbol{\theta} * \mathbf{0.9999991}$. [c] quantile = $\boldsymbol{\theta} * \mathbf{0.9986755}$. [d] quantile = $\boldsymbol{\theta} * \mathbf{0.999963}$.

2 SUPPLEMENTARY NOTE 2: CALIBRATION APPROACH AND DATA SUMMARY

A comprehensive epidemiological calibration dataset was collated to parameterize OpenMalaria. This calibration dataset covers a total of eleven different epidemiological relationships (or objectives for fitting) that span important aspects of the natural history of malaria. Data were collated from different settings (see Supplementary Table 2 for summary) and were detailed in the original model descriptions [2,10] and a later parameterization [1]. A total of 61 simulation scenarios were setup to parameterize OpenMalaria, constructed to simulate the study surveys and study sites that yielded the calibration dataset. The study site observations were replicated in OpenMalaria by reproducing the timing of the surveys and their endpoints (such as prevalence and incidence) and matching simulation options to the setting with regards to transmission intensity and seasonality, vector species, treatment seeking behavior and anti-malarial interventions. The objectives and data are further detailed below.

The parameter estimation process is a multi-objective optimization problem with each of the epidemiological quantities in Supplementary Table 2 representing one objective. The aim of the optimization is to find a parameter set that maximizes the goodness of fit by minimizing a loss statistic computed as the weighted sum of the loss functions for each objective. Building a weighted average reduces the multiple loss terms to a single overall loss statistic, defined as:

$$F(\theta) = \sum_i w_i \sum_j f_{ij}(\theta) \tag{37}$$

where $f_{ij}(\theta)$ is the loss function for parameter vector θ, epidemiological quantity i and dataset j, and the weights w_i were chosen so that different epidemiological quantities contribute approximately equally to $F(\theta)$.

For the current calibration, we utilised the loss functions from Smith et al. 2012 [1], the loss function $f_i(\theta)$ for each objective i use either (negative) log-likelihoods or Residual Sum of Squares (RSS) with an unknown minimum. We did not update these loss-functions in order to compare to our previous approaches.

The likelihood functions are given by

$$\mathcal{L}(\theta|x_1, \dots, x_n) = g(x_1, \dots, x_n|\,\theta) = \prod_{i=1}^{n} g(x_i|\theta) \tag{38}$$

where the observed values are $x_1, \dots, x_n$ and the model parameters θ. In practice, it is easier to work with the log likelihood, namely

$$\log \mathcal{L}(\theta|x_1, \dots, x_n) = \sum_{i=1}^{n} \log g(x_i|\theta) \tag{39}$$

The loss functions $f_i(\theta)$ used for each objective are detailed in the following sections.

Epidemiological quantity	Data sources	No. of scenarios	No. of data points*	Publication for fitting of base model	Prior	Weighting in GOF statistic	Scenario numbers	Loss vector number (f_i)	Loss function
**Age pattern of incidence of infection after intervention**	Molineaux and Gramiccia (1980) [14]	1	12	Maire et al. 2006 [3]	Binomial	0.001	30	1	Binomial log-likelihood
**Age patterns of prevalence of infection**	Molineaux and Gramiccia (1980) [14]	6	563	Maire et al. 2006 [3]	Binomial	0.001	24, 28, 29, 35, 34, 31	2	Binomial log-likelihood
**Age patterns of parasite density**	Molineaux and Gramiccia (1980) [14]	6	563	Maire et al. 2006 [3]	Log Normal	0.01	24, 28, 29, 35, 34, 31	3	log likelihood
**Age pattern of number of concurrent infections**	Maire et al. 2006 [3]; Owusu-Agyei et al. 2002 [15]	1	12	Maire et al. 2006 [3]	Poisson	0.01	34	4	Poisson log-likelihood
**Age pattern of incidence of clinical malaria: age-specific Ndiop & Dielmo, Senegal**	Trape and Rogier 1996 [16]; Kitua et al. 1996 [17]	2	26	Smith et al. 2006 [5]	Log Normal	1	232, 233, 49	5	RSS
**Age pattern of incidence of clinical malaria: infants Idete, Tanzania**	Kitua et al. 1996 [17]	1	4	Smith et al. 2006 [5]	Log Normal	1	49	6	RSS
**Age pattern of threshold parasite density for clinical attacks**	Rogier et al. 1996 [18]	1	13	Smith et al. 2006 [5]	Log Normal	1	234	7	RSS
**Hospitalisation rate in relation to prevalence in children**	Ross et al. 2006 [11]	26	10	Ross et al. 2006 [11]	Log Normal	2	501, 502, 503, 504, 505, 506, 507, 508, 509, 510, 511, 512, 514, 515, 516, 517, 518, 519, 520, 521, 522, 523, 524, 525, 526, 527	8	Squared deviation
**Age pattern of hospitalisation: severe malaria**	Marsh and Snow 1999 [19]	4	12	Ross et al. 2006 [11]	Log Normal	2	158, 167, 173, 176	9	RSS
**Malaria specific mortality in children (<5y)**	Snow et al. 1997 [20]	9	9	Ross et al. 2006 [11]	Log Normal	1	301, 302, 303, 312, 316, 317, 318, 326, 327	10	Squared deviation log Rate
**All-cause infant mortality rate**	Ross et al. 2006 [11]	11	11	Ross et al. 2006 [11]	Log Normal	10	401, 402, 408, 411, 414, 415, 416, 417, 418, 422, 426	11	Squared deviation log Rate

Supplementary Table 2: Epidemiological quantities and data sources used for parameterizing models. (a) Some scenarios are used to predict several outcomes, so the total of this column does not equal the total of 61 scenarios involved in fitting the models. (b) The number of data points is the sum over all scenarios and simulated survey periods of the number of age groups into which the data were disaggregated for comparison with the model predictions. (c) In relation to the EIR specified as a seasonal pattern. (d) Model predictions for this objective are compared with linear interpolations between the field data points. *The number of data points is the sum over all scenarios and simulated survey periods of the number of age groups into which the data were disaggregated for comparison with the model predictions. Table adapted from Table S1 in Smith et al. 2012 [1].

2.1 Objectives: Epidemiological data and loss functions

Below we described each fitting objective in terms of the data (setting, surveys, observations, references) along with the associated loss function and original references. Supplementary Table 3 provides an overview of the 61 simulation scenarios used for calibration, and which objective they contribute to. For space reasons, Supplementary Table 3 is positioned at the end of section 2.1.

2.1.1 Age pattern of incidence after intervention

2.1.1.1 Data

The data used for the calibration of objective 1 (age pattern of incidence) consists of eight cross-sectional surveys of infection rates by age and EIR in Matsari village, capturing 12 age groups each. Matsari village was monitored entomologically for four years (Nov 1970 – Nov 1973) during the Garki Project and multiple anti-malaria interventions were administered [14]. From October 1970 to March 1972 (the baseline / pre-intervention phase), eight cross-sectional malariological surveys of the whole village population and intensive entomologic surveillance (human bait collection of mosquitoes and dissections of the mosquito salivary glands for sporozoites) were carried out. The latter was used to estimate a baseline transmission intensity of 67 inoculations per person per year (EIR) and to derive seasonal transmission patterns. Mid-1972 marked the beginning of the intervention phase, during which an additional eight surveys were carried out at 10-week intervals (surveys 9-16). During this time, indoor residual spraying with Propoxur was carried out comprehensively in the village, along with mass treatment of the population with Sulfadoxine-pyrimethamine at 10 week-intervals immediately after assessment of individuals' parasitologic status. The experimental setup is summarised in Fig. 3 of Smith et al. 2006 [5]. Incidence data (number of patent infections and number of hosts by age) from surveys 9-16 was used for our calibration.

Sites and scenario numbers: Matsari, Nigeria (30)

Original reference detailing data and model fits: Smith TA, Maire N, Dietz K, Killeen GF, Vounatsou P, et al. Relationship between the entomological inoculation rate and the force of infection for Plasmodium falciparum malaria. Am J Trop Med Hyg. Volume 75, No. 2 Supplement. 2006 [5]

2.1.1.2 Loss function: Binomial Log Likelihood

We denote the Binomial log likelihood for this objective to be

$$f_1(\boldsymbol{\theta}) = \log \mathcal{L}(\boldsymbol{\theta}) = \sum_{j=1}^{s}\sum_{k=1}^{a} P_{j,l} \log(\widehat{p_J, k}) + \left(H_{j,k} - P_{j,k}\right) \log(1 - \widehat{p_{J,k}}) \tag{40}$$

where a is the number of age groups, s the number of surveys, $p_{j,k}$ the scenario data number of parasite positive hosts and $H_{j,k}$ the scenario data number of hosts for age group k and survey j. Parameter $\widehat{p_{J,k}}$ is associated with the model predictions and is given by

$$\widehat{p_{J,k}} = \widehat{P_{J,k}} \,/\, \widehat{H_{J,k}} \tag{41}$$

where $\widehat{P_{J,k}}$ are the predicted number of parasite positive hosts and $\widehat{H_{J,k}}$ the predicted number of hosts for age group k and survey j.

2.1.2 Age patterns of prevalence

2.1.2.1 Data

The data used for the calibration of objective 2 (age-patterns of prevalence) consists of six cross-sectional malariology surveys conducted in the Rafin Marke, Matsari, Sugungum villages in Nigeria

1970-1972 (12 age groups each, part of the Garki Project during the pre-intervention period) [14], Navrongo in Ghana 2000 (12 age groups) [15] and Namawala 1990-1991 [21] and Idete in Tanzania (11 and 6 age groups, respectively) 1992-1993 [17]. In all study sites, annual transmission intensity (EIR) and seasonal patterns were assessed using light trap or human night bait collections and dissections of the salivary glands (see Fig. 2 in Maire et al. 2006 [3]). In all sites except Idete, the health system at the time of the surveys treated only a small proportion of the clinical malaria episodes. In the Idete, the village dispensary was assumed to treat approximately 64% of clinical malaria (based on the published literature). During simulation, prevalence was defined by comparing each predicted parasite density with the limit of detection used in the actual study.

Sites and scenario numbers: Sugungum, Nigeria (24); Rafin-Marke, Nigeria (28); Matsari, Nigeria (29); Idete, Tanzania (31); Navrongo, Ghana (34); Namawala, Tanzania (35)

Original reference detailing data and model fits: *Maire N, Smith TA, Ross A, Owusu-Agyei S, Dietz K, et al. A model for natural immunity to asexual blood stages of Plasmodium falciparum malaria in endemic areas. Am J Trop Med Hyg. Volume 75, No. 2 Supplement. 2006* [3]

2.1.2.2 Loss function: Binomial Log Likelihood

We denote the binomial log likelihood for each scenario of this objective to be

$$f_2(\theta) = \log \mathcal{L}(\theta) = \sum_{j=1}^{s}\sum_{k=1}^{a} P_{j,k}\log(p_j,k) + (H_{j,k} - P_{j,k})\log(1 - p_{j,k}) \quad (42)$$

where a is the number of age groups, s the number of surveys, $P_{j,k}$ the scenario data number of parasite positive hosts and $H_{j,k}$ the scenario data number of hosts for age group k and survey j. Parameter $p_{j,k}$ is associated with the model predictions and is given by

$$p_{j,k} = \widehat{P_{J,k}} / \widehat{H_{J,k}} \quad (43)$$

where $\widehat{P_{J,k}}$ are the predicted number of parasite positive hosts and $\widehat{H_{J,k}}$ the predicted number of hosts for age group k and survey j.

2.1.3 Age patterns of parasite density

2.1.3.1 Data

The same data sources as for objective 2 (age pattern of prevalence) were used for calibration of objective 3 (age pattern of parasite density). Parasite densities in sites that were part of the Garki project (Sugungum, Rafin-Make and Matsari, Nigeria) were recorded by scanning a predetermined number of microscope fields on the thick blood film and recording how many had one or more asexual parasites visible. These were converted to numbers of parasites visible by assuming Poisson distribution for the number of parasites per field and a blood volume of 0.5 mm^3 per 200 fields. In the other studies (Idete and Namawala, Tanzania and Navrongo, Ghana), parasites were counted against leukocytes and converted to nominal parasites/microliter assuming the usual standard of 8,000 leukocytes/microliter. The biases in density estimates resulting from these different techniques were accounted for by multiplying the observed parasite densities with constant values estimated for Garki (ν_0) and non-Garki (ν_1) studies to rescale them to the values in malariatherapy patients [22].

Sites and scenario numbers: Sugungum, Nigeria (pre-intervention, 24); Rafin-Marke, Nigeria (pre-intervention, 28); Matsari, Nigeria (pre-intervention, 29); Idete, Tanzania (31); Navrongo, Ghana (34); Namawala, Tanzania (35)

Original reference detailing data and model fits: Maire N, Smith TA, Ross A, Owusu-Agyei S, Dietz K, et al. A model for natural immunity to asexual blood stages of Plasmodium falciparum malaria in endemic areas. Am J Trop Med Hyg. Volume 75, No. 2 Supplement. 2006 [3]

2.1.3.2 Loss function: Log-normal log likelihood

For objective 3 (age pattern of parasite densities) we denote the log-Normal log likelihood for each scenario to be

$$f_3(\theta) = \log \mathcal{L}(\theta) = n(\log(\rho) - \log(\sigma)) - 0.5\mathrm{RSS}/\sigma^2 \quad (44)$$

where n is the number of observations in the data set, $\rho = \exp(-0.5\log(2\,\pi))$, a constant from the log-normal likelihood, RSS is the residual sum of squares given by

$$\mathrm{RSS} = \sum_{j=1}^{s}\sum_{k=1}^{a}\left(\frac{\widehat{Y_{j,k}}}{\widehat{P_{j,k}}} - \log(\nu) - \frac{Y_{j,k}}{P_{j,l}}\right)^2 \quad (45)$$

and σ is the standard deviation given by

$$\sigma = \sqrt{\mathrm{RSS}/\,(n-1)} \quad (46)$$

Here, ν is the appropriate density bias, which is a fitting parameter, a is the number of age groups, s is the number of surveys, $P_{j,k}$ the scenario number of parasite positive hosts, and $Y_{j,k}$ the sum of the log densities, $\widehat{P_{j,k}}$ the predicted number of parasite positive hosts and $\widehat{Y_{j,k}}$ the predicted sum of the log densities for age group k and survey j. The density bias are fitting parameters ν_0 and ν_1.

2.1.4 Age pattern of number of concurrent infections

2.1.4.1 Data

For objective 4 (age pattern of number of concurrent infections), the dataset from Navrongo, Ghana (also used in the calibration of objectives 2 and 3) was used to calibrate to the total numbers of distinct parasite infections in one individual in each age group, and at each survey. Distinct infections were detected by polymerase chain reaction-restriction fragment length polymorphism in the sampled individuals.

Sites and scenario numbers: Navrongo, Ghana (34)

Original reference detailing data and model fits: Maire N, Smith TA, Ross A, Owusu-Agyei S, Dietz K, et al. A model for natural immunity to asexual blood stages of Plasmodium falciparum malaria in endemic areas. Am J Trop Med Hyg. Volume 75, No. 2 Supplement. 2006 [3]

2.1.4.2 Loss function: Poisson Log Likelihood

Assuming that both the data and the simulations are Poisson distributed about the correct value and thereby also allowing for over-dispersion, we denote the Poisson log likelihood for each scenario to be for the objective of age pattern of number of concurrent infections to be

$$f_4(\theta) = \log \mathcal{L}(\theta) = \sum_{j=1}^{s}\sum_{k=1}^{a} -Pn_{j,k}\log\left(Pn_{j,k}\,/\,\lambda_{j,k}\right) + Pn_{j,k} - \lambda_{j,k} \quad (47)$$

where a is the number of age groups, s the number of surveys, $Pn_{j,k}$ the scenario data total patent infections for age group k and survey j. Parameter $\lambda_{j,k}$ is associated with the model predictions and is given by

$$\lambda_{j,k} = \frac{\widehat{Pn_{J,k}}}{\widehat{H_{J,k}}} H_{j,k} \tag{48}$$

where $\widehat{Pn_{J,k}}$ are the predicted total of patent infections and $\widehat{H_{J,k}}$ the predicted number of hosts for age group k and survey j and $H_{j,k}$ is the scenario data number of hosts for age group k and survey j.

2.1.5 Age pattern of incidence of clinical malaria

2.1.5.1 Data

Two distinct datasets representing three study sites (Supplementary Table 4) were used for the calibration of objective 5 and objective 6 (age pattern of incidence of clinical malaria). For Objective 5, the dataset contains data on the age pattern of clinical episodes in the villages of Ndiop and Dielmo in Senegal [16,23]. During the study period of July 1990 - June 1992, the village populations were visited daily to detect and treat any clinical malaria attacks with quinine. Cases were detected by reporting of symptoms (fever) during daily active case detection and subsequent thick blood smear microscopy. Only symptomatic individuals (axillary temperature ≥ 38.0°C or rectal temperature ≥ 38.5°C). Due to the active case detection and rapid treatment, all symptomatic episodes are assumed to be effectively treated in these villages during the study period. No effective treatment of clinical malaria was assumed prior to the study period. The annual patterns of transmission were replicated as reported by Charlwood et al. (1998) [24]. A proportion P_t=35.75% are assumed to be treated effectively in Idete. As all individuals reporting to the village dispensary were treated presumptively with chloroquine, this proportion corresponds to the proportion of episodes reported to the village dispensary.

Sites and scenario numbers: Ndiop, Senegal (232), Dielmo, Senegal (233)

Original reference detailing data and model fits: Smith TA, Ross A, Maire N, Rogier C, Trape J-F, et al. An epidemiologic model of the incidence of acute illness in Plasmodium falciparum malaria. Am J Trop Med Hyg. Volume 75, No. 2 Supplement. 2006 [10]

2.1.5.2 Loss function: RSS-biased

We denote a loss function based on biased residual sum of squares:

$$f_5(\theta) = \sum_{j=s_1}^{s} \sum_{k=1}^{a} R^2 \tag{49}$$

where a is the number of age groups, s the number of surveys, s_1 the initial survey number, and R is the residual given by

$$R = I_{i,j} - \frac{\widehat{C_{J,k}}}{\left(\widehat{H_{J,k}}\right)} \frac{1}{\mu} \tag{50}$$

where $I_{j,k}$ is the observed recorded incidence rate, $\widehat{C_{J,k}}$ are the predicted total cases (severe and uncomplicated), $\widehat{H_{J,k}}$ the predicted number of hosts for age group k and survey j and μ is a bias related to the scenario. For scenarios 232 and 233 (representing Ndiop and Dielmo, Senegal) this bias is $\mu = 5$ indicating the duration in years for which episodes are collected. For scenario 49 in Objective 6 (Idete, Tanzania) the bias is $\mu = 0.357459$ and represents the proportion of episodes reported to the village dispensary.

Scenario No.	Study site	Age groups	Observations
**232**	Ndiop, Senegal	22	One per age group
**233**	Dielmo, Senegal	22	One per age group
**49**	Idete, Tanzania	4	One per age group

Supplementary Table 4: Summary of study data set for objective 5: Age pattern of incidence of clinical malaria.

2.1.6 Age pattern of incidence of clinical malaria: infants

2.1.6.1 Data

Objective 6 (age pattern of incidence of clinical malaria in infants) is informed by a dataset on incidence that contains passive case detection data on the age-incidence in infants recorded at the health centre in Idete, Tanzania from June 1993-October 1994 [17]. The annual patterns of transmission were replicated as reported by Charlwood et al. (1998) [24].

Sites and scenario numbers: Idete, Tanzania (49))

Original reference: Smith TA, Ross A, Maire N, Rogier C, Trape J-F, et al. An epidemiologic model of the incidence of acute illness in Plasmodium falciparum malaria. Am J Trop Med Hyg. Volume 75, No. 2 Supplement. 2006 [10]

2.1.6.2 Loss function: RSS-biased

The loss function for Objective 6 is the same as Objective 5. For scenario 49 (Idete, Tanzania) the bias is $\mu = 0.357459$ and represents the proportion of episodes reported to the village dispensary.

2.1.7 Age pattern of threshold parasite density for clinical attacks

2.1.7.1 Data

Objective 7 (age pattern of threshold parasite density for clinical attacks) uses the dataset from Dielmo, Senegal (see objective 5) for calibration. The pyrogenic threshold in the (OpenMalaria) predictions is output as the sum of the log threshold values across age groups. The pyrogenic threshold per age group is given as the parasite:leukocyte ratio for recorded incidence of disease. To adjust these densities to the same scale as that used in fitting the simulation model to other datasets, the parasite:leukocyte ratios were multiplied by a factor of 1,416 to give a notional density in parasites/microliter of blood. This number was derived as follows: Parasites were counted against leukocytes and converted to nominal parasites/microliter assuming the usual (though biased) standard of 8,000 leukocytes/microliter. The biases in density estimates resulting from these different techniques was accounted for by multiplying the observed parasite densities with constant values estimated for Garki (ν_0) and non-Garki (ν_1) studies to rescale them to the values in malariatherapy patients [22].The value 1,416 comes from

$$8000\nu_1 \tag{51}$$

where the original $\nu_1 \approx 0.18$.

Sites and scenario numbers: Dielmo, Senegal (234)

Original reference detailing data and model fits: Smith TA, Ross A, Maire N, Rogier C, Trape J-F, et al. An epidemiologic model of the incidence of acute illness in Plasmodium falciparum malaria. Am J Trop Med Hyg. Volume 75, No. 2 Supplement. 2006 [10]

2.1.7.2 Loss function: RSS-biased (log)

For the objective 7 (Age pattern of threshold parasite density for clinical attacks) we denote a residual sum of squares loss function given by (13) with

$$f_7(\theta) = \log\left(Y^*_{j,k}\right) - \frac{\widehat{Y^*_{J,k}}}{\widehat{H_{J,k}}} - \log(\mu) \qquad (52)$$

where Y^* is the observed pyrogenic threshold, $\widehat{Y^*}$ are the predicted sum log pyrogenic threshold, $\widehat{H_{J,k}}$ the predicted number of hosts for age group k and survey j and is a bias related to the scenario. Here, this bias is related to the log parasite/leucocyte ratio and thus $\mu = 1/(8000\nu_1)$ where ν_1 is the non-Garki density bias.

2.1.8 Hospitalization rate in relation to prevalence in children

2.1.8.1 Data

Data on the relative incidence of severe malaria-related morbidity and mortality in children <9 years old across different transmission intensities were originally collated by Marsh and Snow (1999) [19] (Table 4). Data measurements per age group were available as the relative risk of severe disease compared to age group 1 and the proportion/prevalence of severe episodes. A total of 26 entries on the relationship between severe malaria hospital admission rates and *P. falciparum* prevalence were used to calibrate objective 8 (Hospitalisation rate in relation to prevalence in children), each represented in a separate simulation scenario, with one observation per scenario. These are summarised in Supplementary Table 5. To obtain a continuous function relating hospital incidence rates to prevalence, linear interpolation between data points was performed. To convert hospital incidence rates to community severe malaria incidence, the hospital admission rates was divided by the assumed proportion of severe episodes representing to hospital (48%). There was assumed to be no effective treatment of uncomplicated malaria episodes or malaria mortality.

Sites and scenario numbers: Bo, Sierra Leone (501); Niakhar, Senegal (502), Farafenni, The Gambia (503); Areas I-V, The Gambia (504-508); Gihanga, Burundi (509); Katumba, Burundi (510); Karangasso, Burkina Faso (511); Kilifi North, Kenya (512); Manhica, Mozambique (514); Namawala, Tanzania (515); Navrongo, Ghana (516); Saradidi, Kenya (517); Yombo, Tanzania (518); Ziniare, Burkina Faso (519); Matsari, Nigeria (520); ITC control, Burkina Faso (521); Mlomp, Senegal (522); Ganvie, Benin (523); Kilifi Town, Kenya (524); Chonyi, Kenya (525); Bandafassi, Senegal (526); Kongodjan, Burkina Faso (527)

Original reference detailing data and model fits: Ross A, Maire N, Molineaux L, and Smith TA. An epidemiologic model of severe morbidity and mortality caused by Plasmodium falciparum. Am J Trop Med Hyg. Volume 75, No. 2 Supplement. 2006 [11]

2.1.8.2 Loss function: squared deviation

The loss function is denoted as the log of residual sum of squares

$$f_8(\theta) = \left[\log\left(\frac{a_s\widehat{R_{k=1}}}{R^*_{k=1}}\right)\right]^2 \qquad (53)$$

where a_s is the access to treatment of severe cases (0.48, estimated in base model), $\widehat{R_{k=1}}$ is the scenario predicted rate of severe episodes per 1000 person-years for age group $k = 1$ (0-9 years), and parameter $R^*_{k=1}$ is the interpolated observed rate of severe episodes per 1000 person year given by

$$R^*_{k=1} = \frac{\left(\widehat{P_{k=1}} - P_l\right)}{(P_u - P_l)}(R_u - R_l) + R_t \tag{54}$$

where $\widehat{P_{k=1}}$ is the predicted prevalence summed over all surveys, P_u and P_l are the observed prevalences above and below the predicted prevalence $\widehat{P_{k=1}}$, respectively and R_u and R_l are the corresponding severe episode rates to the observed prevalences.

The predicted prevalence is given by

$$\widehat{P_{k=1}} = \frac{\widehat{Pt_{k=1}}/24}{\widehat{H_{k=1}}/24} \tag{55}$$

where $\widehat{P_{k=1}}$ is the total number of parasite positive predicted and $\widehat{H_{k=1}}$ are the total number of hosts (division by 24 to give mean values). The predicted rate of episodes per 1000 person year is given by

$$\widehat{R_{k=1}} = \frac{1000\,\widehat{S_{k=1}/2}}{\widehat{H_{k=1}/24}} \tag{56}$$

where $\widehat{S_{k=1}}$ is the number of severe cases predicted and with division by 2 to convert to from 2 years to 1 year and the division by 24 to give mean number of hosts.

Site		EIR data	
		**Year**	**EIR**
**Burkina Faso**			
	ITC Control	1994-1995	389
	Karangasso	1985	244
	Kongodjan	1984	133
	Ziniare	1994-1995	70
**Burundi**			
	Gihanga	1983	205
	Katumba	1982	13.6
**Kenya**			
	Chonyi	1992-1993	50
	Kilifi North	1992-1003	10.5
	Kilifi Town	1990-1991	2.8
	Saradidi	1986-1987	239
**Senegal**			
	Bandafassi	1995-1996	363
	Mlomp	1995	30
	Niakhar	1995	11.6
**Tanzania**			
	Namawala	1990-1991	329
	Yombo	1992	234
**The Gambia**			
	Area I-V	1991	+
	Farafenni	1987	8.9
**Others**			
	Bo, Sierra Leone	1990-1991	34.7
	Ganvie, Benin	1993-1995	11
	Manhica, Mozambique	2001-2002	38
	Matsari, Nigeria	1971	68
	Navrongo, Ghana	2001-2002	418

Supplementary Table 5: Settings used for calibrating the incidence of severe malaria. (Adapted from Table1 from Ross et al. 2006 [11])

*EIR = entomological inoculation rate, ITC = control group of randomised trial of insecticide-treated curtains. +Five sites with annual EIR between 1 and 10

2.1.9 Age pattern of hospitalization: severe malaria

2.1.9.1 Data

For objective 9 (Age pattern of hospitalisation), a subset of the data collated by Marsh and Snow (1999) [19] (see objective 8) is used. Detailed age-specific severe hospital admission rates were available for 5 of the sites (Supplementary Table 6). The patterns of incidence by age were summarised by age in 1-4 and 5-9 year-old children and compared with 1-11 month old infants by calculating the relative risk. Of the five sites, four were selected for fitting objective 9 based on the predicted prevalence. Baku, The Gambia was excluded as the very low (2%) prevalence here could not be matched.

Sites and scenario number(s): Area V, The Gambia (158); Saradidi, Kenya (167); Ganvie, Benin (173); Bandafassi, Senegal (176)

Original reference detailing data and model fits: Ross A, Maire N, Molineaux L, and Smith TA. An epidemiologic model of severe morbidity and mortality caused by Plasmodium falciparum. Am J Trop Med Hyg. Volume 75, No. 2 Supplement. 2006 [11]

Estimate	Sukuta, The Gambia	Kilifi North, Kenya	Kilifi South, Kenya	Siaya, Kenya
Years of paediatric ward surveillance	1992-95	1990-95	1992-96	1992,1994-96
Person-years exposure to risk of children aged 0-9 yr	23,468	52,675	45,967	40,064
**Rates**				
All-cause malaria, age 1-11 mo	23.3 (17.8–28.9) [66/2830]	59.5 (53.2-65.9) [318/5342]	79.9 (71.6-86.4) [407/5152]	84.6 (76.4-92.8) [374/4420]
All-cause malaria, age 1-4 yr	35.3 (32.2-39.4) [372/10379]	41.7 (39.0-44.4) [905/21714]	17.4 (15.5-19.3) [321/18493]	18.8 (16.7-20.9) [312/16567]
All-cause malaria, age 5-9 yr	16.3 (13.8-18.8) [167/10259]	5.3 (4.4-6.2) [135 / 25619]	1.7 (1.2-2.2) [38/22322]	1.7 (1.1-2.3) [33/19077]
All-cause malaria, age 0-9 yr	25.8 (23.8-27.8) [605]	25.9 (24.5-27.2) (1363)+	16.7 (15.5-17.9) [766]	18.0 (16.7-19.3) [719]
Cerebral malaria$ 0-9 yr	2.6 (2.0-3.3) [61]	1.5 (1.2-1.8) [79]	0.8 (0.5-1.1) [36]	0.1 (0.0-0.2) [5]
Severe malaria anaemia, § 0-9 yr	NA	5.0 (4.4-5.6) [262]	4.2 (3.6-4.8) [192]	3.7 (2.7-4.7) [50/13416]
All-cause acute respiratory tract admissions age 0-9 yr	8.4 (7.3-9.6) [198]	9.3 (8.5-10.1) [492]	8.3 (7.5-9.1) [380]	8.7 (7.8-9.6) [348]

Supplementary Table 6: Age-specific period prevalence rates* of severe malaria, severe malaria, severe malaria anaemia and acute respiratory-tract infections from five communities in The Gambia and Kenya. (Adapted from Table 2 from Snow et al. 1997 [20])

* Period prevalence rather than incidence because precise matching of each community member to hospital admission was not possible. Rates as admission per 1000 children per year (95% CI). +Precise dates of birth were unobtainable for five children. $Defined as child admitted with primary diagnosis of malaria and Blantyre coma score of 2 or less. § Defined in child with primary diagnosis of malaria and haemoglobin of 5.0g/dL or less on admission. Rates for Siaya derived from person-years exposure to risk and admissions for period Nov 1, 1994 to Oct 31, 1995.

2.1.9.2 Loss function: Residual sums of squares for relative risk

We denote a loss function based on residual sum of squares:

$$f_9(\theta) = \sum_{k=2,3} \left[\log \frac{\widehat{RR}_k}{RR_k}\right]^2 \tag{57}$$

where RR_k is the relative risk of severe episode for age group k compared to age group 1 and $\widehat{RR_k}$ is the predictive relative risk for age group k compared to age group 1. The predicted relative risk is given by

$$\widehat{RR_k} = \frac{\widehat{S_k}}{\widehat{H_k}} - \frac{\widehat{S_1}}{\widehat{H_1}} \tag{58}$$

where $\widehat{S_k}$ is the number of severe cases predicted for age group k and $\widehat{H_k}$ the total number of hosts for age group k.

2.1.10 Malaria specific mortality in children (< 5 years old)

2.1.10.1 Data

For objective 10 (Malaria specific mortality in children <5 years old), mortality data were derived from verbal autopsy studies in sites with prospective demographic surveillance and were adjusted for the effect of malaria transmission intensity on the sensitivity and specificity of the cause of death determination [25]. The data are provided in Supplementary Table 7. The odds ratio for death of a case in the community relative to that in hospital was estimated by fitting to the malaria-specific mortality rates in children less than five years of age assuming the published hospital case fatality rate. Nine sites for which both malaria-specific mortality rates and seasonal transmission patterns were available were included for calibration.

There is one observation per study site and simulation scenario, and predicted values are for one survey at the end of 2 years.

Sites and scenario number(s): Bo, Sierra Leone (301); Niakhar, Senegal (302); Farafenni, The Gambia (303); Kilifi North, Kenya (312); Navrongo, Ghana (316); Saradidi, Kenya (317); Yombo, Tanzania (318); Bandafassi, Senegal (326); Kongodjan, Burkina Faso (327)

Location	EIR	Mortality (95% CI)	Person-years observed	Malaria deaths observed
Bo, Sierra Leone	34.7	12.8 (4.8-21.2)	776	9
Niakhar, Senegal	11.6	10.9 (6.9-15.6)	29,491	307
Farafenni, The Gambia	8.9	9.4 (4.8-14.6)	3,130	29
Kilifi North, Kenya	10.5	9.2 (5.9-12.9)	20,679	155
Navrongo, Ghana	417	9.3 (1.9-17.0)	3,815	55
Saradidi, Kenya	239	20.8 (12.8-29.8)	8,035	142
Yombo, Tanzania	234	22.1 (14.0-33.1)	5,850	54
Bandafassi, Senegal	363	5.9 (2.8-9.3)	8,488	50
Kongodjan, Burkina Faso	133	2.2 (0-5.2)	1271	3

Supplementary Table 7: Direct malaria mortality data in children <5 years old used for fitting OpenMalaria. Table adapted from Table 2 in [11] and Table 1 in [25].

Original reference detailing data and model fits: Ross A, Maire N, Molineaux L and Smith TA. An epidemiologic model of severe morbidity and mortality caused by Plasmodium falciparum. Am J Trop Med Hyg. Volume 75, No. 2 Supplement. 2006 [11]

2.1.10.2 *Loss function: Residual sums of squares*

For objective 10 on Malaria specific mortality in children, the loss function minimizes the log sum of squares

$$f_{10}(\boldsymbol{\theta}) = \left[\log\left(\frac{\widehat{DMR_1}}{DMR_1}\right)\right]^2 \tag{59}$$

where DMR_1 is the observed direct mortality rate for age group 1 (0-5 years) and $\widehat{DMR_1}$ is the predicted direct mortality rate for age group 1. The predicted direct mortality rate is given by

$$\widehat{DMR_1} = \frac{\widehat{DD_1}}{2\widehat{H_1}} \tag{60}$$

where $\widehat{DD_1}$ is the number of direct malaria deaths cases predicted for age group 1 and $\widehat{H_1}$ the total number of predicted hosts for age group 1. The division by 2 is to convert to yearly rate as the survey was conducted at the end of 2 years.

2.1.11 Indirect malaria infant mortality rate

2.1.11.1 Data

For objective 11 (indirect malaria infant mortality rate), a subset of the data collated by Marsh and Snow (1999) [19] (see objective 8) was used. These constitute a library of sites for which entomologic data were collected at least monthly and all-cause infant mortality rates (IMR) were available. There is one observation per scenario: all cause infant mortality rate (returned as a single number over whole intervention period).

Sites and scenario number(s): Bo, Sierra Leone (401); Niakhar, Senegal (402); Area V, The Gambia (408); Karangasso, Burkina Faso (411); Manhica, Mozambique (414); Namawala, Tanzania (415); Navrongo, Ghana (416); Saradidi, Kanya (417); Yombo, Tanzania (418); Mlomp, Senegal (422); Bandafassi, Senegal (426)

Original reference detailing data and model fits: Ross A, Maire N, Molineaux L, and Smith TA. An epidemiologic model of severe morbidity and mortality caused by Plasmodium falciparum. Am J Trop Med Hyg. Volume 75, No. 2 Supplement. 2006 [11]

2.1.11.2 Loss function: Residual sums of squares

The loss function minimises the log sum of squares:

$$f_{11}(\theta) = \left[\log\left(\frac{i\widehat{DMR_1}}{iDMR_1}\right)\right]^2 \tag{61}$$

where $iDMR_1$ the observed indirect mortality rate for age group 1 and $i\widehat{DMR_1}$ is the predicted indirect mortality rate for age group 1.

2.2 Supplementary Table 3

Scen. No.	Site/reference	Description	Objective(s)	Data reference
**24**	Sugungum, Nigeria (pre-intervention phase)	8 cross sectional surveys of entire village population at 10-week intervals (4,487 blood slides)	Age-prevalence (2);<br>Age-parasite densities (3)	Molineaux and Gramiccia. 1980 [14]
**28**	Rafin-Marke, Nigeria (pre-intervention phase)	8 cross sectional surveys of entire village population at 10-week intervals (2,593 blood slides)	Age-prevalence (2);<br>Age-parasite densities (3)	Molineaux and Gramiccia. 1980 [14]
**29**	Matsari, Nigeria (pre-intervention phase)	8 cross sectional surveys of entire village population at 10-week intervals (2,963 blood slides)	Age-prevalence (2);<br>Age-parasite densities (3)	Molineaux and Gramiccia. 1980 [14]
**30**	Matsari, Nigeria (intervention phase)	8 cross sectional surveys of entire village population at 10-week intervals (2,663 blood slides)	Age-incidence of patent infections (1)	Molineaux and Gramiccia. 1980 [14]
**31**	Idete, Tanzania	Surveillance of a rolling cohort of infants (1,382 blood slides over 16 months). Also 1 cross-sectional survey of 312 children 1-5 months	Age-prevalence (2);<br>Age-parasite densities (3)	Kitua et al. 1996 [17]
**34**	Navrongo, Ghana	6 age-stratified cross-sectional surveys at 2-month intervals (total 522 slides / DNA samples)	Age-prevalence (2);<br>Age-parasite densities (3),<br>Age-specific multiplicity of infection (4)	Owusu-Agyei S et al. 2002 [15]
**35**	Namawala, Tanzania	12 age-stratified cross-sectional surveys at 2-month intervals (3,901 blood slides)	Age-prevalence (2);<br>Age-parasite densities (3)	Smith et al. 1993 [21]
**49**	Idete, Tanzania	Passive case detection at the village dispensary over 15 months in 12 age groups.	Age Pattern of Incidence of Clinical Malaria in Idete in infants (6)	Kitua et al. 1996 [17]; Vounatsou et al. 2000 [26]
**158**	Area V, The Gambia	Hospitalisation rate by age	Age pattern of severe hospitalisation (9)	Snow et al. 1997 [20]
**167**	Saradidi, Kenya	21 cohorts each of approximately 50 children between 6 months and 6 years of age whose parasites were cleared and who were then followed up with 2 weekly surveys. Hospitalisation rate by age.	Age pattern of severe hospitalisation (9)	Beier et al. 1999 [27], Snow 1997 [20]
**173**	Ganvie, Benin	Hospitalisation rate by age.	Age pattern of severe hospitalisation (9)	Snow et al. 1997 [20]

Scen. No.	Site/reference	Description	Objective(s)	Data reference
**176**	Bandafassi, Senegal	Hospitalisation rate by age.	Age pattern of severe hospitalisation (9)	Snow et al. 1997 [20]
**232**	Ndiop, Senegal	Longitudinal study of 350 permanent residents over 2 years: Individual level active case detection three times a week (questionnaire + recording of symptoms) and parasitological surveys twice a week; daily recording of new fever cases at compound level. By age group (9 groups)	Age pattern of incidence of clinical malaria (6)	Trape JF and Rogier C. 1996 [16]
**233**	Dielmo, Senegal	Longitudinal study of 206 permanent residents over 2 years: Individual level active case detection three times a week (questionnaire + recording of symptoms) and parasitological surveys twice a week; daily recording of new fever cases at compound level. By age group (9 groups)	Age pattern of incidence of clinical malaria by age (6)	Trape JF and Rogier C. 1996 [16]
**234**	Dielmo, Senegal	Longitudinal study of 206 permanent residents over 2 years: Individual level active case detection three times a week (questionnaire + recording of symptoms) and parasitological surveys twice a week; daily recording of new fever cases at compound level. By age group (9 groups)	Age Pattern of parasite density threshold for clinical attack (7)	Trape JF and Rogier C. 1996 [16]
**301**	Bo, Sierra Leone	Point estimate based on a 1-year longitudinal study covering 776 person-years	Direct Malaria Mortality (10)	Korenromp et al. 2003 [25]
**302**	Niakhar, Senegal	Point estimate based on 5-year longitudinal study covering 29,491 person-years [XML label: Diohine]	Direct Malaria Mortality (10)	Korenromp et al. 2003 [25]
**303**	Farafenni, The Gambia	Point estimate based on 2-year longitudinal study covering 2,263 person-years [XML label: Tally Ya]	Direct Malaria Mortality (10)	Korenromp et al. 2003 [25]
**312**	Kilifi North, Kenya	Point estimate based on 3-year longitudinal study covering 20,679 person-years	Direct Malaria Mortality (10)	Korenromp et al. 2003[25]
**316**	Navrongo, Ghana	Point estimate based on 1-year longitudinal study covering 1,065 person-years	Direct Malaria Mortality (10)	Korenromp et al. 2003 [25]
**317**	Saradidi, Kenya	21 cohorts each of approximately 50 children between 6 months and 6 years of age whose parasites were cleared and who were then followed up with 2 weekly surveys.	Direct Malaria Mortality (10)	Korenromp et al. 2003 [25]

Scen. No.	Site/reference	Description	Objective(s)	Data reference
**318**	Yombo, Tanzania	Point estimate based on 3-year longitudinal study covering 5,850 person-years	Direct Malaria Mortality (10)	Korenromp et al. 2003 [25]
**326**	Bandafassi, Senegal	Point estimate based on 6-year longitudinal study covering 8,488 person-years	Direct Malaria Mortality (10)	Korenromp et al. 2003 [25]
**327**	Kongodjan, Burkina Faso	Point estimate based on 5-year longitudinal study covering 1,271 person-years	Direct Malaria Mortality (10)	Korenromp et al. 2003 [25]
**401**	Bo, Sierra Leone	Point estimates of all-cause neonatal, post-neonatal, and infant mortality rates	All-cause mortality (11)	Barnish et al. 1993 [28]
**402**	Niakhar, Senegal	Point estimates of all-cause neonatal, post-neonatal, and infant mortality rates; XML label: Diohine	All-cause mortality (11)	INDEPTH Network, 2002 [29]; Spencer et al. 1987 [30]
**408**	Area V, The Gambia	Point estimates of all-cause neonatal, post-neonatal, and infant mortality rates	All-cause mortality (11)	D'Alessandro et al. 1995 [31]
**411**	Karangasso, Burkina Faso	Point estimates of all-cause neonatal, post-neonatal, and infant mortality rates	All-cause mortality (11)	Duboz et al. 1989 [32]
**414**	Manhica, Mozambique	Point estimates of all-cause neonatal, post-neonatal, and infant mortality rates	All-cause mortality (11)	INDEPTH Network, 2002 [29]
**415**	Namawala, Tanzania	Point estimates of all-cause neonatal, post-neonatal, and infant mortality rates; Pre-intervention	All-cause mortality (11)	Armstrong-Schellenberg et al. 1999 [33]
**416**	Navrongo, Ghana	Point estimates of all-cause neonatal, post-neonatal, and infant mortality rates	All-cause mortality (11)	INDEPTH Network, 2002 [29]
**417**	Saradidi, Kanya	21 cohorts each of approximately 50 children between 6 months and 6 years of age whose parasites were cleared and who were then followed up with 2 weekly surveys.	All-cause mortality (11)	Spencer et al. 1987[30]
**418**	Yombo, Tanzania	Point estimates of all-cause neonatal, post-neonatal, and infant mortality rates	All-cause mortality (11)	Premji Z et al. 1997 [34]
**422**	Mlomp, Senegal	Point estimates of all-cause neonatal, post-neonatal, and infant mortality rates	All-cause mortality (11)	Trape et al. 1998 [35]
**426**	Bandafassi, Senegal	Point estimates of all-cause neonatal, post-neonatal, and infant mortality rates	All-cause mortality (11)	INDEPTH Network, 2002 [29]
**501**	Bo, Sierra Leone	Point estimate of the severe malaria hospital admission rate and *P. falciparum* prevalence in children <9 years old.	Severe episodes by prevalence (8)	Marsh and Snow 1999 [19]

Scen. No.	Site/reference	Description	Objective(s)	Data reference
**502**	Niakhar, Senegal	Point estimate of the severe malaria hospital admission rate and *P. falciparum* prevalence in children <9 years old.XML label: Diohine (ca 20 km from Niakhar)	Severe episodes by prevalence (8)	Marsh and Snow 1999 [19]
**503**	Farafenni, The Gambia	Point estimate of the severe malaria hospital admission rate and *P. falciparum* prevalence in children <9 years old.XML label: Tally Ya (ca 15 km from Farafenni)	Severe episodes by prevalence (8)	Marsh and Snow 1999 [19]
**504**	Area I, The Gambia	Point estimate of the severe malaria hospital admission rate and *P. falciparum* prevalence in children <9 years old.	Severe episodes by prevalence (8)	Marsh and Snow 1999 [19]
**505**	Area II, The Gambia	Point estimate of the severe malaria hospital admission rate and *P. falciparum* prevalence in children <9 years old.	Severe episodes by prevalence (8)	Marsh and Snow 1999 [19]
**506**	Area III, The Gambia	Point estimate of the severe malaria hospital admission rate and *P. falciparum* prevalence in children <9 years old.	Severe episodes by prevalence (8)	Marsh and Snow 1999 [19]
**507**	Area IV, The Gambia	Point estimate of the severe malaria hospital admission rate and *P. falciparum* prevalence in children <9 years old.	Severe episodes by prevalence (8)	Marsh and Snow 1999 [19]
**508**	Area V, The Gambia	Point estimate of the severe malaria hospital admission rate and *P. falciparum* prevalence in children <9 years old.	Severe episodes by prevalence (8)	Marsh and Snow 1999 [19]
**509**	Gihanga, Burundi	Point estimate of the severe malaria hospital admission rate and *P. falciparum* prevalence in children <9 years old.	Severe episodes by prevalence (8)	Marsh and Snow 1999 [19]
**510**	Katumba, Burundi	Point estimate of the severe malaria hospital admission rate and *P. falciparum* prevalence in children <9 years old.	Severe episodes by prevalence (8)	Marsh and Snow 1999 [19]
**511**	Karangasso, Burkina Faso	Point estimate of the severe malaria hospital admission rate and *P. falciparum* prevalence in children <9 years old.	Severe episodes by prevalence (8)	Marsh and Snow 1999 [19]
**512**	Kilifi North, Kenya	Point estimate of the severe malaria hospital admission rate and *P. falciparum* prevalence in children <9 years old.	Severe episodes by prevalence (8)	Marsh and Snow 1999 [19]
**514**	Manhica, Mozambique	Point estimate of the severe malaria hospital admission rate and *P. falciparum* prevalence in children <9 years old.	Severe episodes by prevalence (8)	Marsh and Snow 1999 [19]

Scen. No.	Site/reference	Description	Objective(s)	Data reference
**515**	Namawala, Tanzania	Point estimate of the severe malaria hospital admission rate and *P. falciparum* prevalence in children <9 years old. Pre-intervention	Severe episodes by prevalence (8)	Marsh and Snow 1999 [19]
**516**	Navrongo, Ghana	Point estimate of the severe malaria hospital admission rate and *P. falciparum* prevalence in children <9 years old.	Severe episodes by prevalence (8)	Marsh and Snow 1999 [19]
**517**	Saradidi, Kenya	Point estimate of the severe malaria hospital admission rate and *P. falciparum* prevalence in children <9 years old.	Severe episodes by prevalence (8)	Marsh and Snow 1999 [19]
**518**	Yombo, Tanzania	Point estimate of the severe malaria hospital admission rate and *P. falciparum* prevalence in children <9 years old.	Severe episodes by prevalence (8)	Marsh and Snow 1999 [19]
**519**	Ziniare, Burkina Faso	Point estimate of the severe malaria hospital admission rate and *P. falciparum* prevalence in children <9 years old.	Severe episodes by prevalence (8)	Marsh and Snow 1999 [19]
**520**	Matsari, Nigeria	Point estimate of the severe malaria hospital admission rate and *P. falciparum* prevalence in children <9 years old. Pre-intervention	Severe episodes by prevalence (8)	Marsh and Snow 1999 [19]
**521**	ITC control, Burkina Faso	Point estimate of the severe malaria hospital admission rate and *P. falciparum* prevalence in children <9 years old.	Severe episodes by prevalence (8)	Marsh and Snow 1999 [19]
**522**	Mlomp, Senegal	Point estimate of the severe malaria hospital admission rate and *P. falciparum* prevalence in children <9 years old.	Severe episodes by prevalence (8)	Marsh and Snow 1999 [19]
**523**	Ganvie, Benin	Point estimate of the severe malaria hospital admission rate and *P. falciparum* prevalence in children <9 years old.	Severe episodes by prevalence (8)	Marsh and Snow 1999 [19]
**524**	Kilifi Town, Kenya	Point estimate of the severe malaria hospital admission rate and *P. falciparum* prevalence in children <9 years old.	Severe episodes by prevalence (8)	Marsh and Snow 1999 [19]
**525**	Chonyi, Kenya	Point estimate of the severe malaria hospital admission rate and *P. falciparum* prevalence in children <9 years old.	Severe episodes by prevalence (8)	Marsh and Snow 1999 [19]

Scen. No.	Site/reference	Description	Objective(s)	Data reference
**526**	Bandafassi, Senegal	Point estimate of the severe malaria hospital admission rate and *P. falciparum* prevalence in children <9 years old.	Severe episodes by prevalence (8)	Marsh and Snow 1999 [19]
**527**	Kongodjan, Burkina Faso	Point estimate of the severe malaria hospital admission rate and *P. falciparum* prevalence in children <9 years old.	Severe episodes by prevalence (8)	Marsh and Snow 1999 [19]

Supplementary Table 3: Calibration data for objectives 2-4, age patterns of prevalence, parasite densities, and multiplicity of infection

3 EMULATOR PERFORMANCE

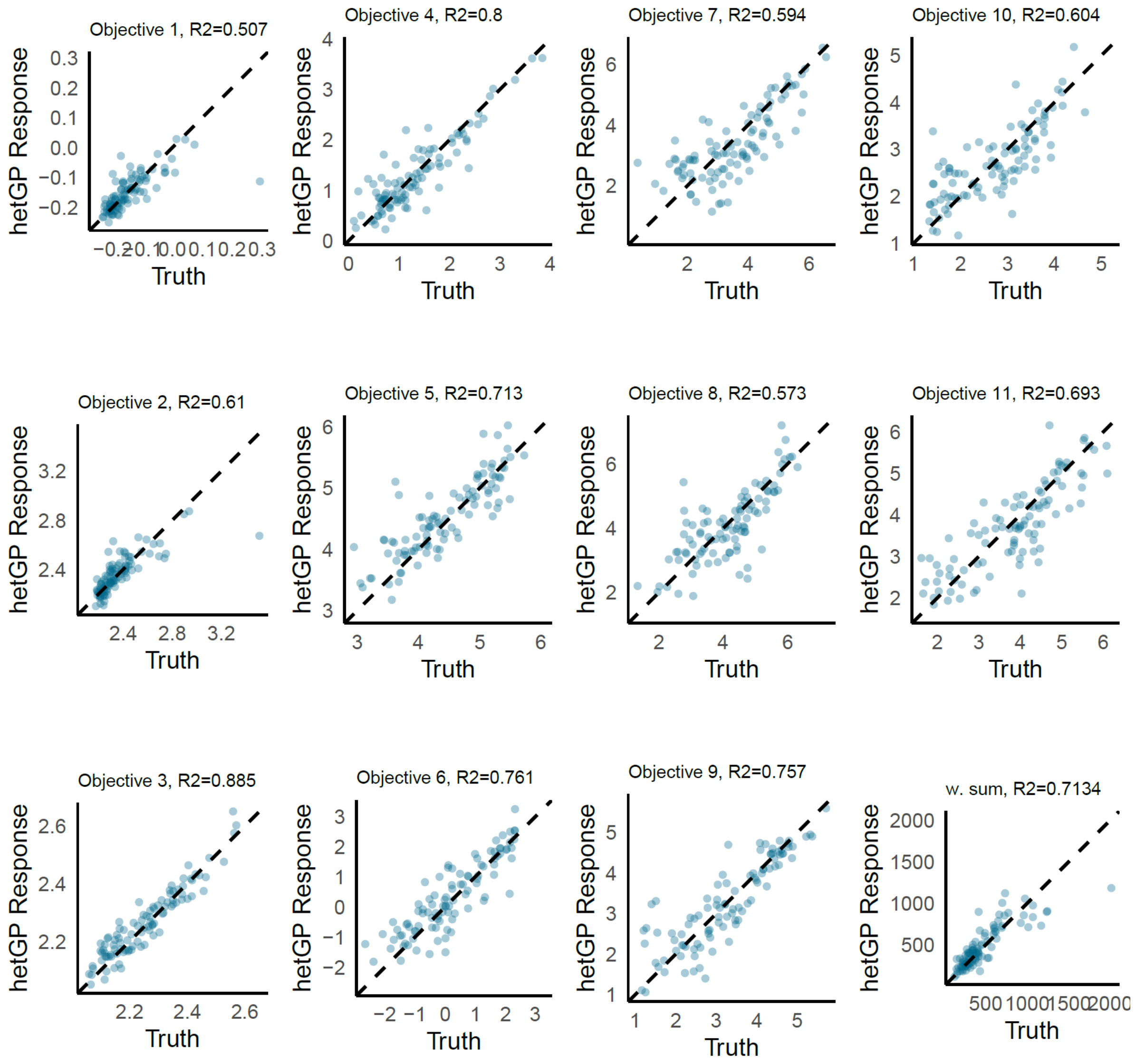

Supplementary Figure 2. GP emulator performance. Emulator predictions vs true values on a holdout set compromising 10% of initial samples in iteration 1. w.sum is the weighted sum F, of the 11 objectives. Here, predictions are generated as the weighted sum of individual objective predictions.

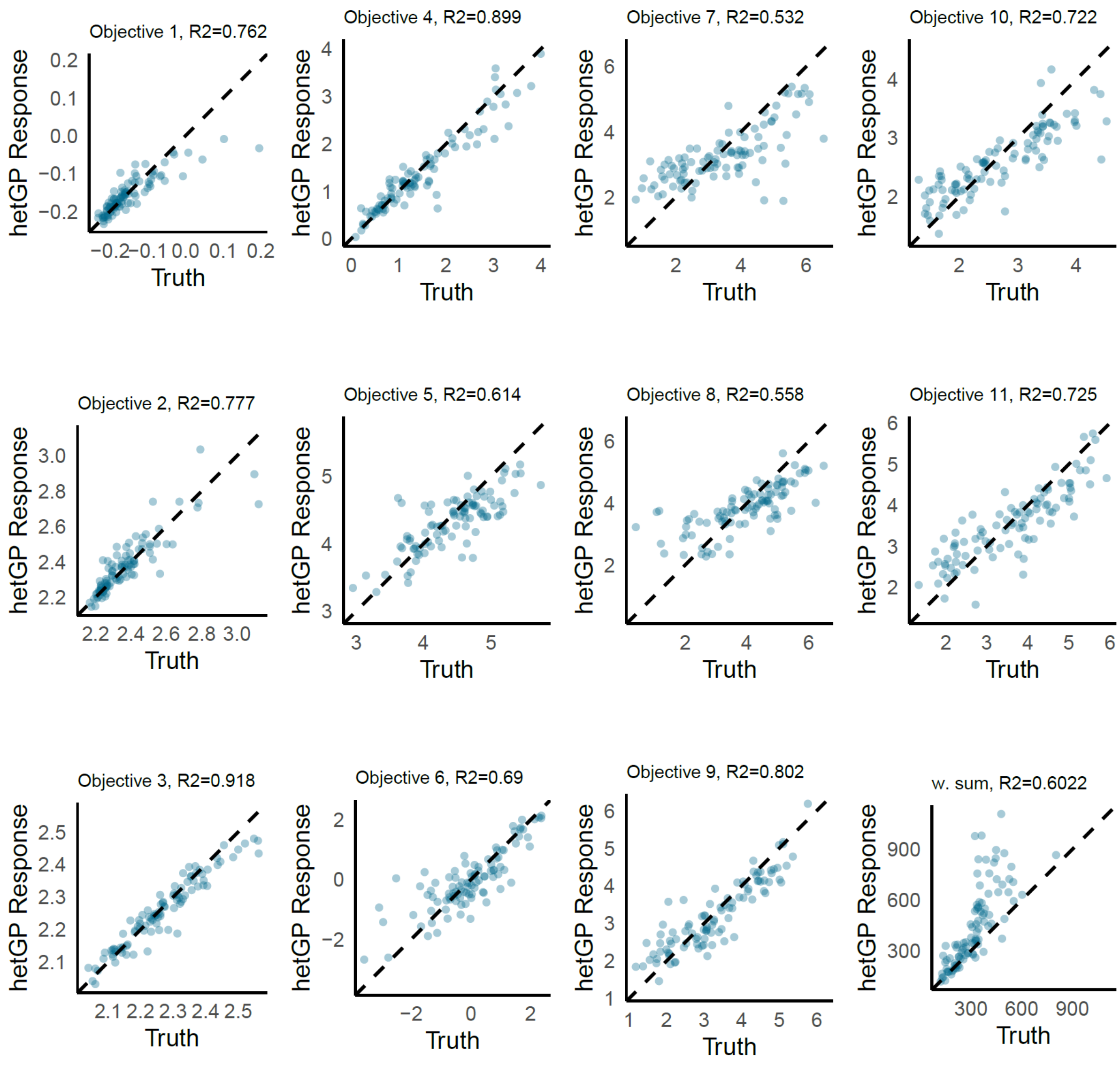

Supplementary Figure 3. GP emulator performance. Emulator predictions vs true values on a holdout set compromising 10% of initial samples in iteration 30 (final iteration). w.sum is the weighted sum F, of the 11 objectives. Here, predictions are generated as the weighted sum of individual objective predictions.

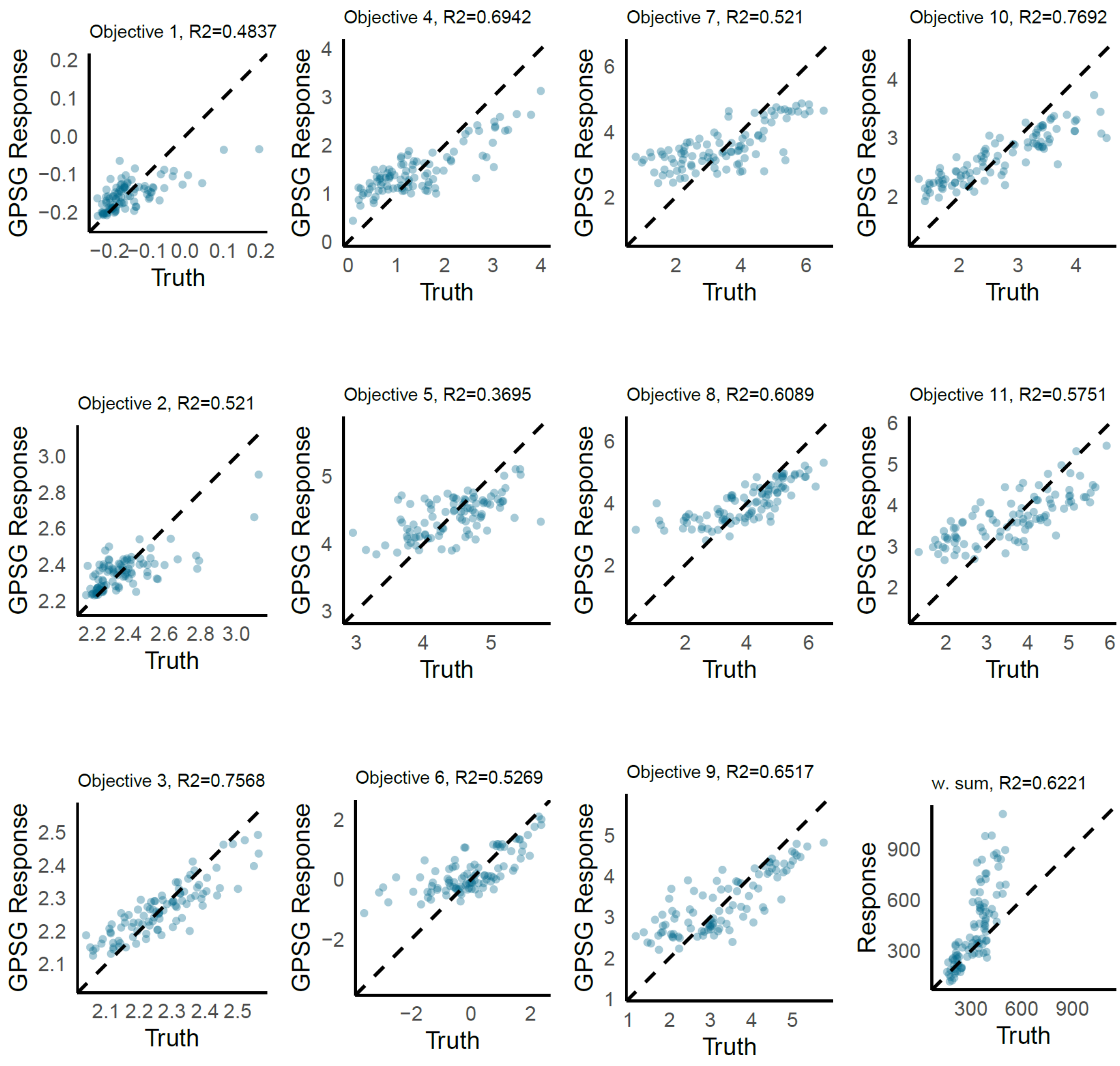

Supplementary Figure 4. GPSG emulator performance. Emulator predictions vs true values on a holdout set compromising 10% of initial samples in iteration 1. w.sum is the weighted sum F, of the 11 objectives. Here, predictions are generated as the weighted sum of individual objective predictions.

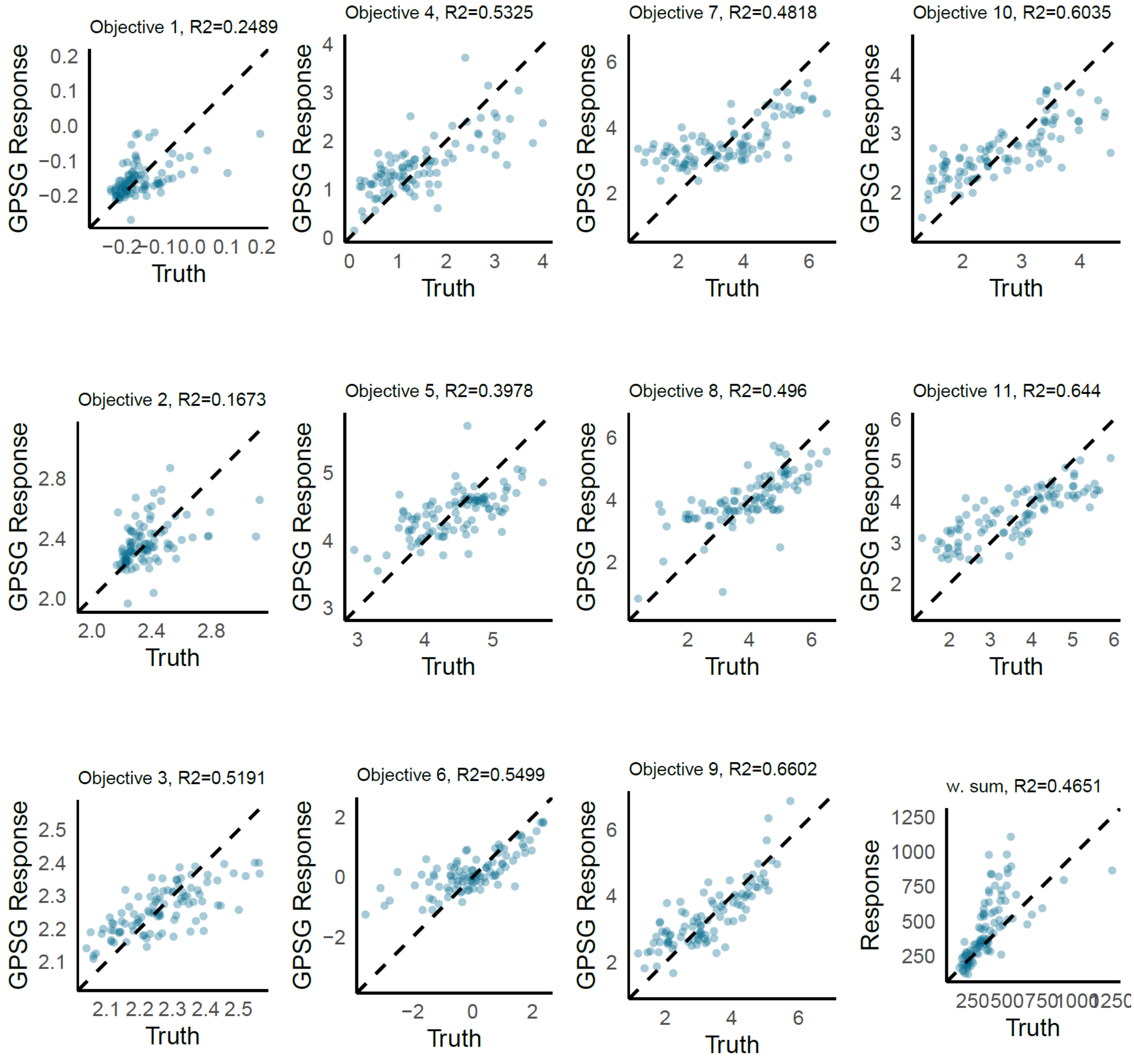

Supplementary Figure 5. GPSG emulator performance. Emulator predictions vs true values on a holdout set compromising 10% of initial samples in iteration 23 (final iteration). w.sum is the weighted sum F, of the 11 objectives. Here, predictions are generated as the weighted sum of individual objective predictions.

4 ADAPTIVE SAMPLING: SELECTED POINTS

4.1 GP-BO

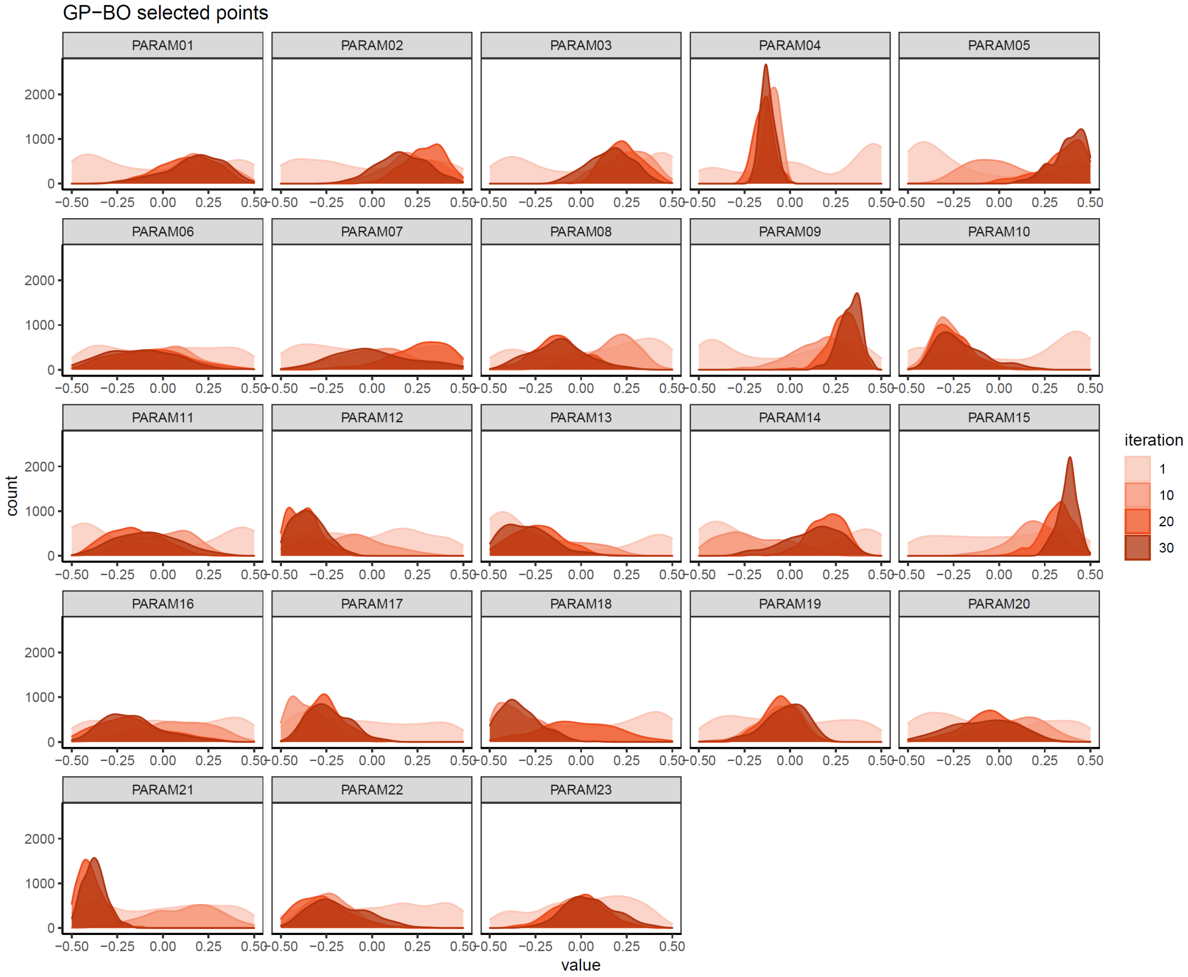

Supplementary Figure 6. GP-BO sampling behavior. Values in each dimension of the points sampled during adaptive sampling of GP-BO algorithm in iterations 1,10, 20, and 30. Plot labels represent parameter numbers (“PARAMXX”, where “XX” refers to the parameter number).

4.2 GPSG-BO

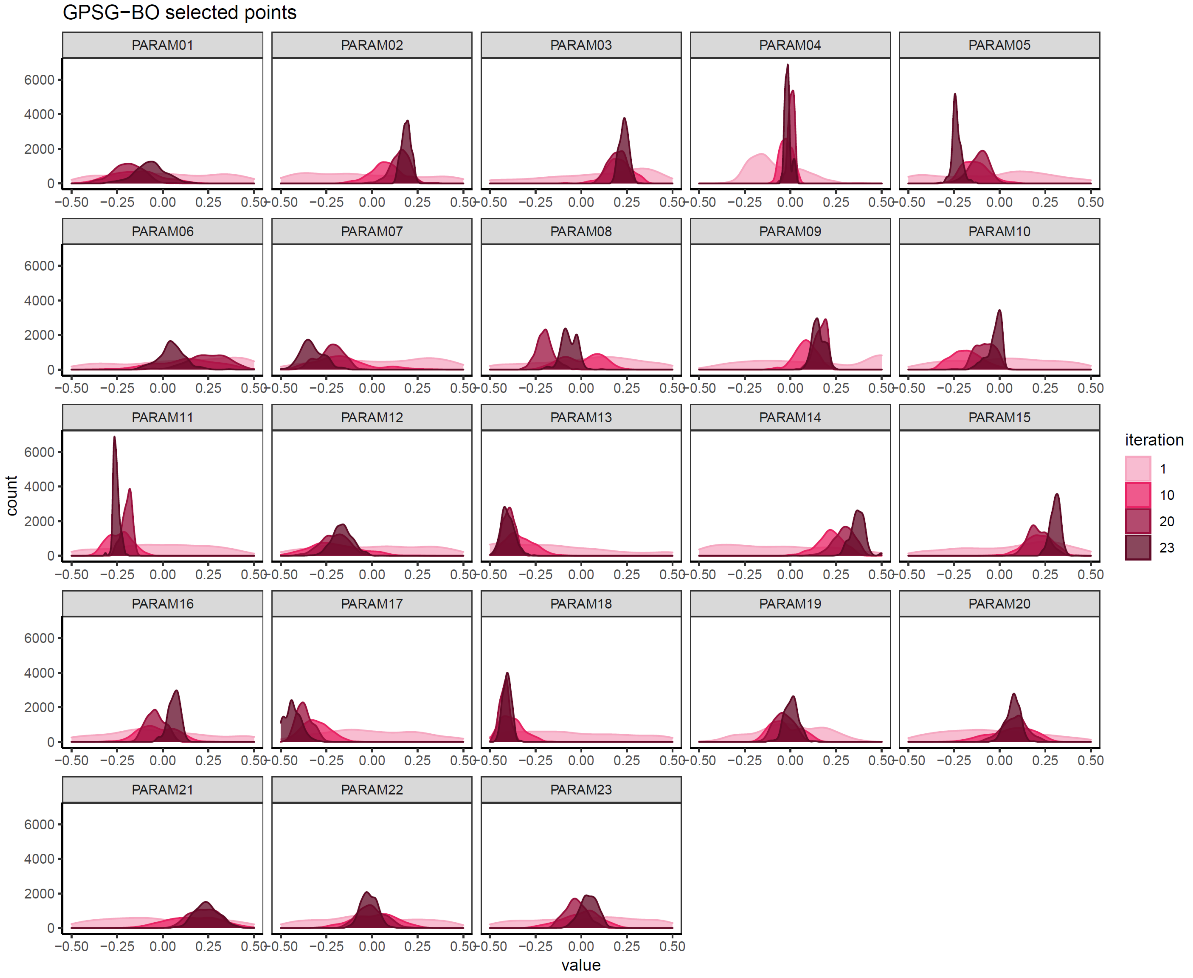

Supplementary Figure 7. GPSG-BO sampling behavior. Values in each dimension of the points sampled during adaptive sampling of GPSG-BO algorithm in iterations 1,10, 20, and 23. Plot labels represent parameter numbers (“PARAMXX”, where “XX” refers to the parameter number).

5 OPENMALARIA: FINAL SIMULATOR FIT

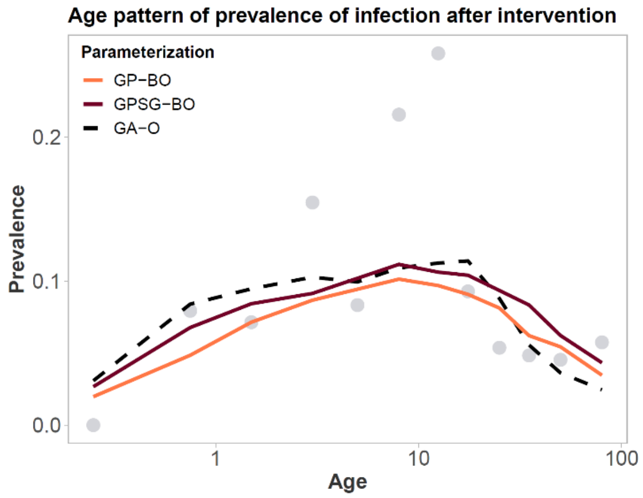

Supplementary Figure 8. Objective 1: Age pattern of prevalence in Matsari, Nigeria during the intervention. Final simulator fit using the parameter sets yielded using GP-BO (orange line) and GPSG-BO (red line) compared to the previous parameterization (derived using optimization with a genetic algorithm, GA-O, black dashed line). Grey dots show the calibration data.

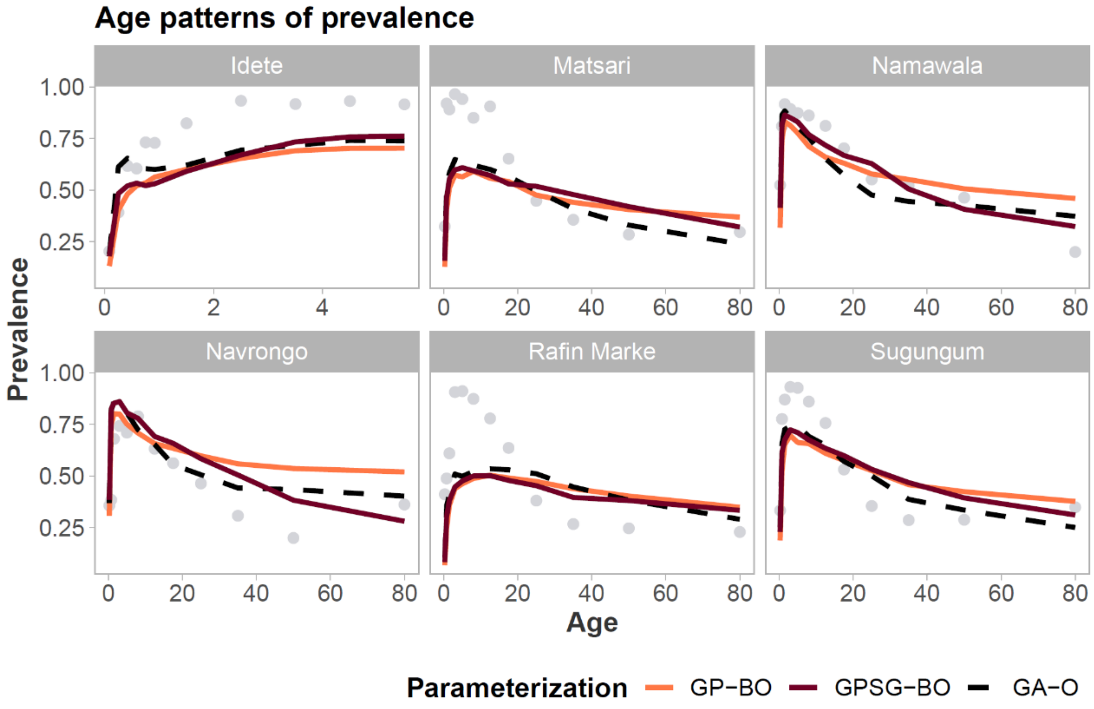

Supplementary Figure 9. Objective 2: Age pattern of prevalence. Final simulator fit using the parameter sets yielded using GP-BO (orange line) and GPSG-BO (red line) compared to the previous parameterization (derived using optimization with a genetic algorithm, GA-O, black dashed line). Grey dots show the calibration data.

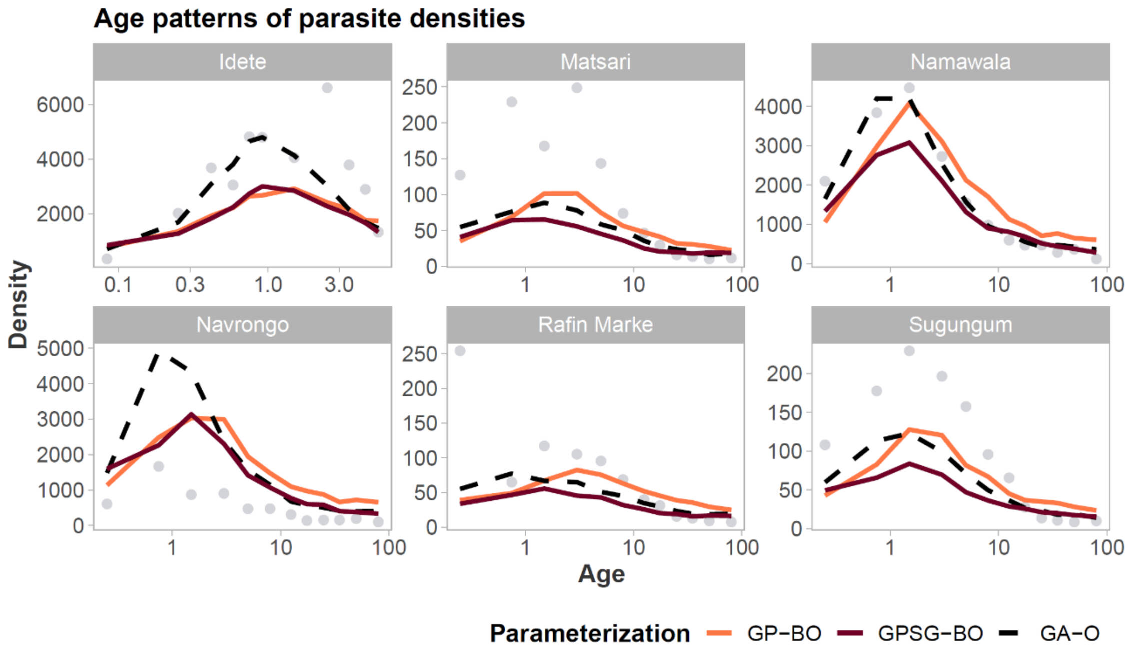

Supplementary Figure 10. Objective 3: **Age pattern of parasite densities (geometric mean).** Final simulator fit using the parameter sets yielded using GP-BO (orange line) and GPSG-BO (red line) compared to the previous parameterization (derived using optimization with a genetic algorithm, GA-O, black dashed line). Grey dots show the calibration data.

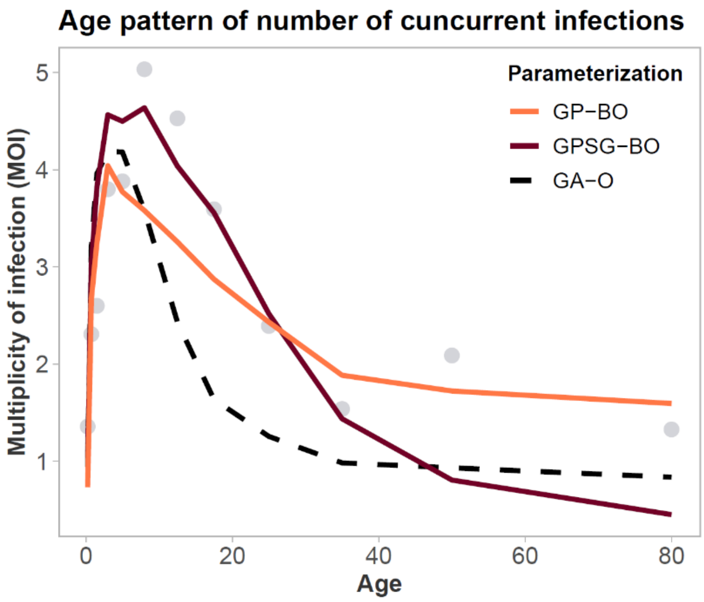

Supplementary Figure 11. Objective 4: Age pattern of number of concurrent infections. Final simulator fit using the parameter sets yielded using GP-BO (orange line) and GPSG-BO (red line) compared to the previous parameterization (derived using optimization with a genetic algorithm, GA-O, black dashed line). Grey dots show the calibration data.

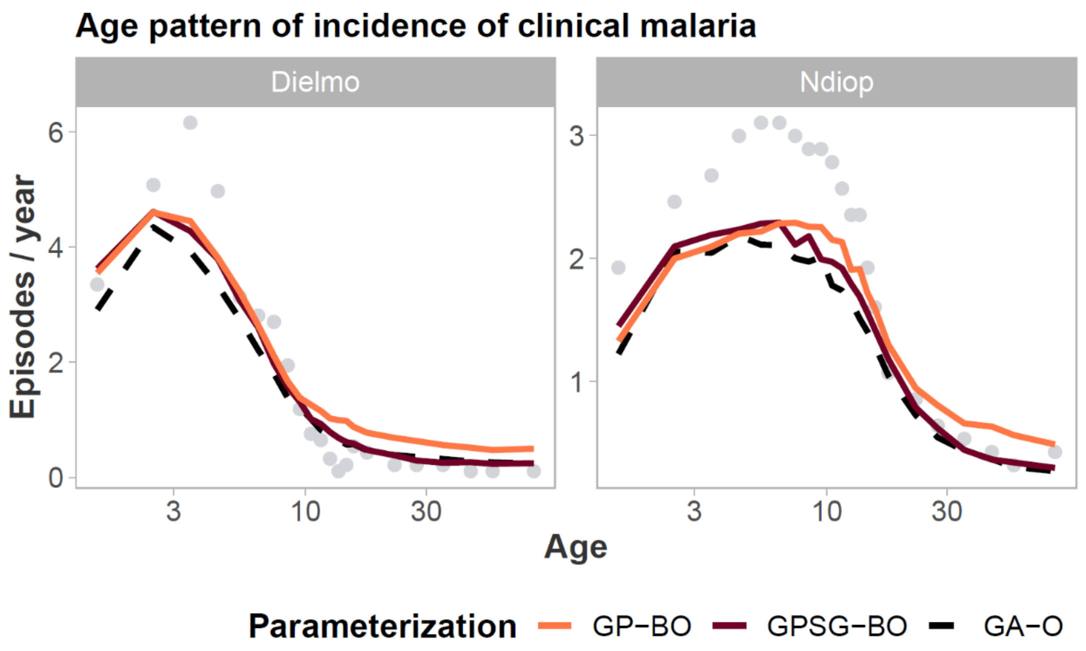

Supplementary Figure 12. Objective 5: Age pattern of incidence of clinical malaria in Dielmo and Ndiop, Senegal. Final simulator fit using the parameter sets yielded using GP-BO (orange line) and GPSG-BO (red line) compared to the previous parameterization (derived using optimization with a genetic algorithm, GA-O, black dashed line). Grey dots show the calibration data.

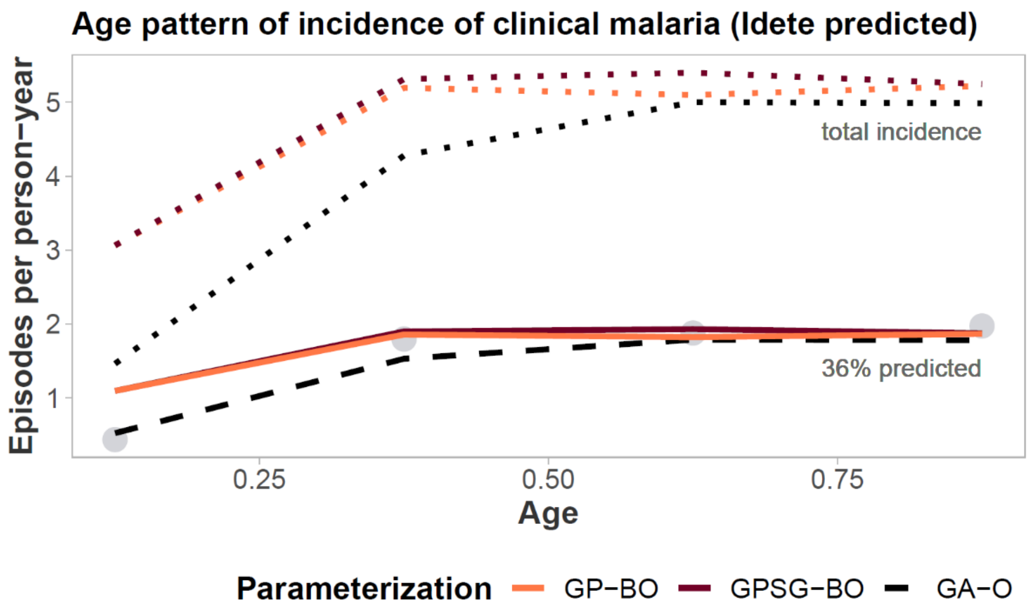

Supplementary Figure 13. Objective 6: Age pattern of incidence of clinical malaria in Idete, Tanzania. Final simulator fit using the parameter sets yielded using GP-BO (orange lines) and GPSG-BO (red lines) compared to the previous parameterization (derived using optimization with a genetic algorithm, GA-O, black lines). Dotted lines show the total incidence and solid lines (dashed for GA-O) show the 36% of the total incidence recorded in the health system. Grey dots show the calibration data.

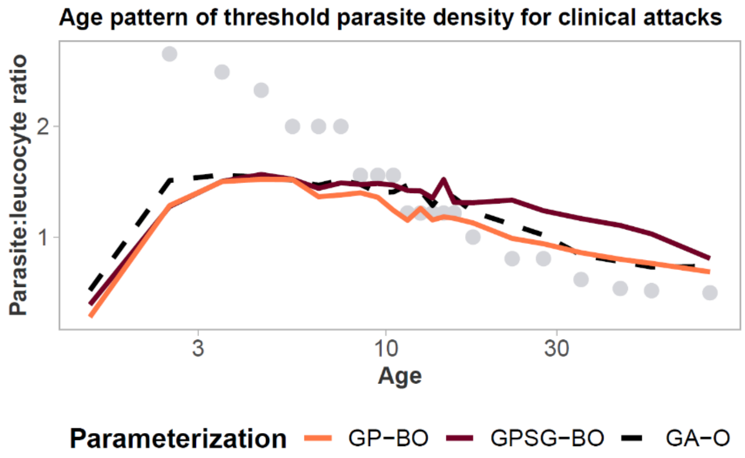

Supplementary Figure 14. Objective 7. Age pattern of threshold parasite density for clinical attacks. Final simulator fit using the parameter sets yielded using GP-BO (orange line) and GPSG-BO (red line) compared to the previous parameterization (derived using optimization with a genetic algorithm, GA-O, black dashed line). Grey dots show the calibration data.

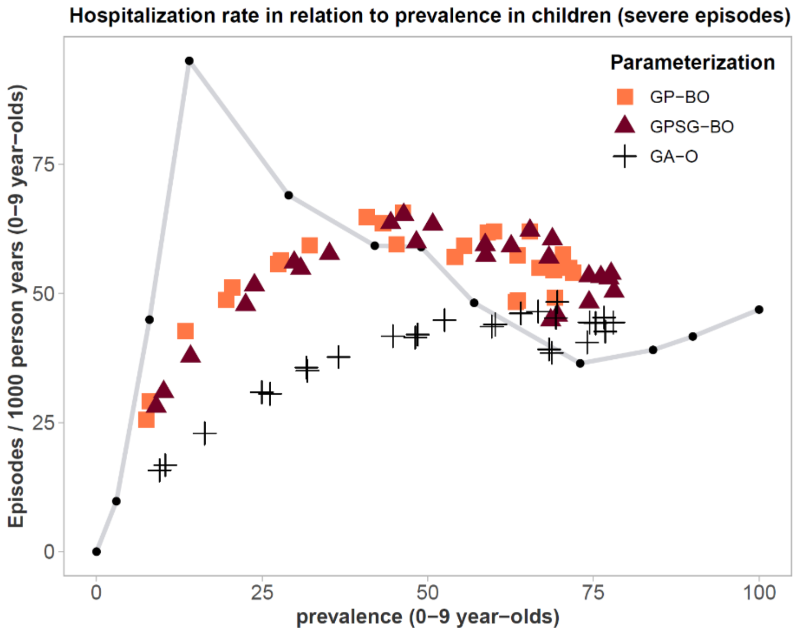

Supplementary Figure 15. Objective 8: Hospitalization rate in relation to prevalence in children. Final simulator fit using the parameter sets yielded using GP-BO (orange squares) and GPSG-BO (red triangles) compared to the previous parameterization (derived using optimization with a genetic algorithm, GA-O, black crosses). Black dots and the grey line show the calibration data.

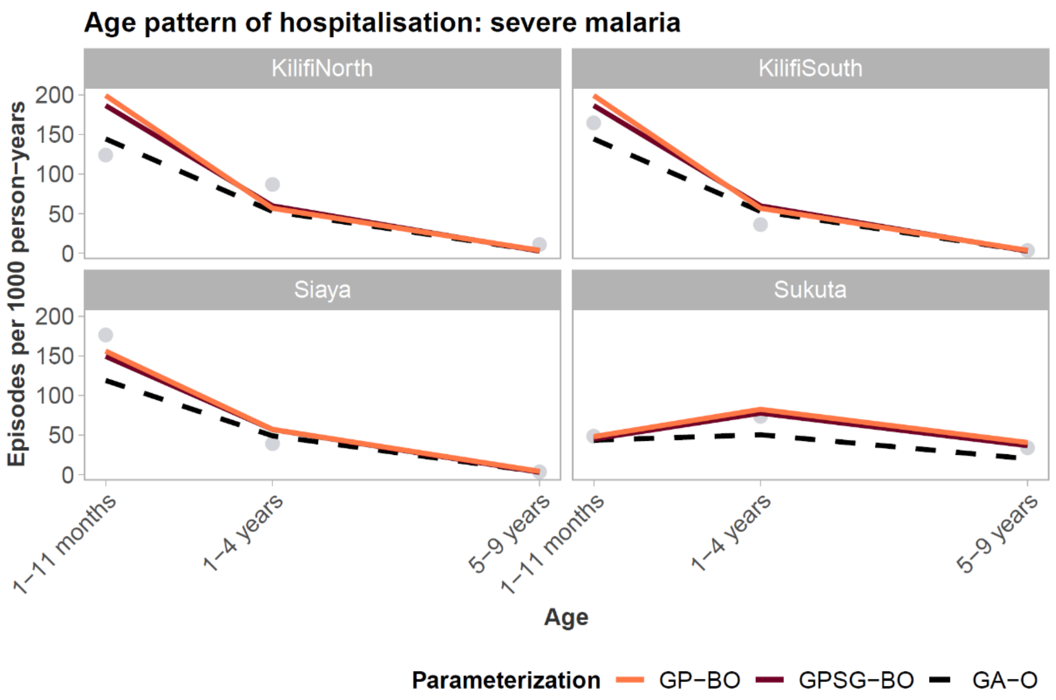

Supplementary Figure 16. Objective 9. Age pattern of hospitalization. Final simulator fit using the parameter sets yielded using GP-BO (orange line) and GPSG-BO (red line) compared to the previous parameterization (derived using optimization with a genetic algorithm, GA-O, black dashed line). Grey dots show the calibration data.

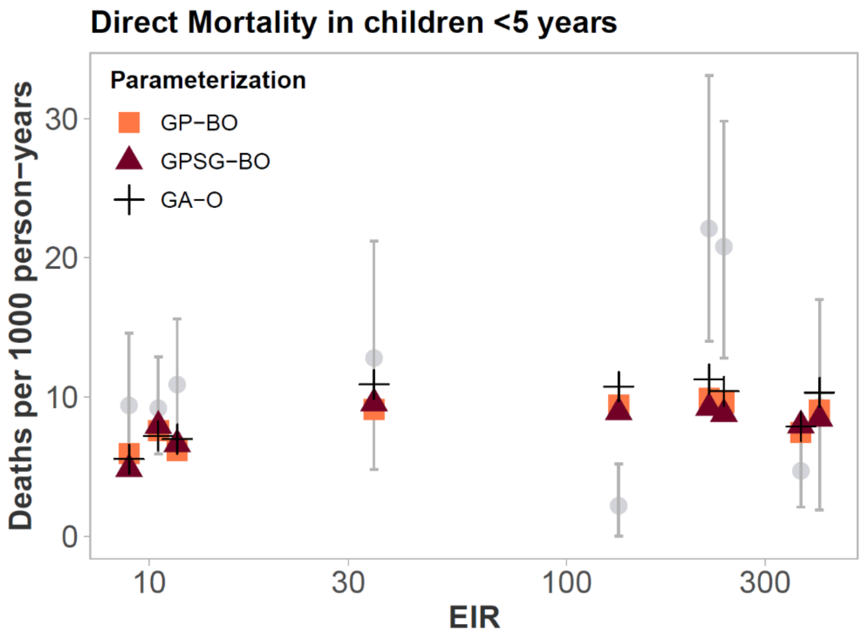

Supplementary Figure 17. Objective 10: Direct mortality in children <5 years old. Final simulator fit using the parameter sets yielded using GP-BO (orange squares) and GPSG-BO (red triangles) compared to the previous parameterization (derived using optimization with a genetic algorithm, GA-O, black crosses). Grey dots and error bars show the calibration data (see Supplementary Table 7), representing median estimates and 95% confidence intervals (samples sizes from left to right 3130, 20679, 29491, 776, 1271, 5850, 8035, 8488, and 3815 person-years observed).

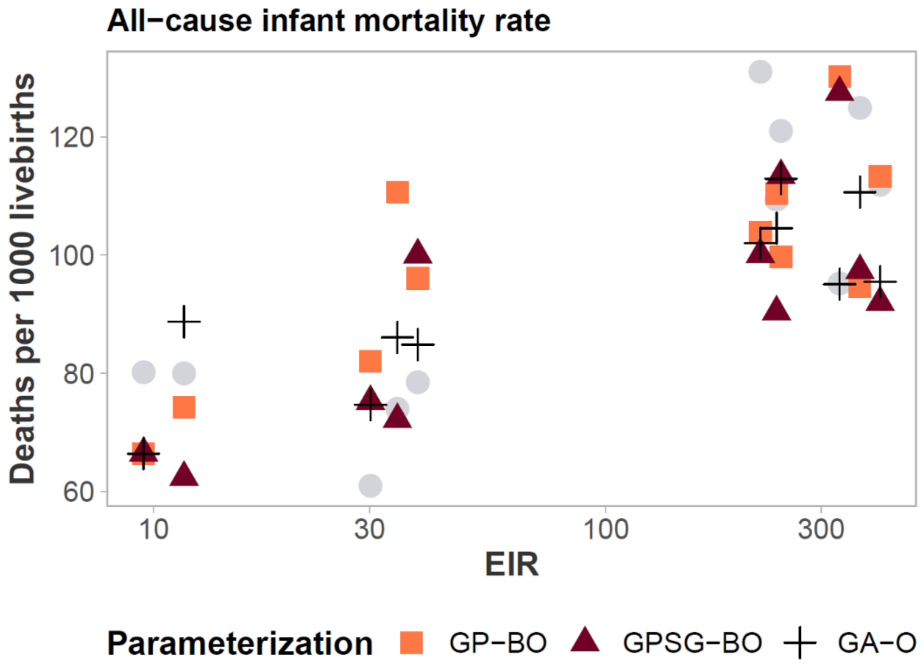

Supplementary Figure 18. Objective 11: All-cause infant mortality rate. Final simulator fit using the parameter sets yielded using GP-BO and GPSG-BO compared to the previous parameterization (derived using optimization with a genetic algorithm, GA-O). Final simulator fit using the parameter sets yielded using GP-BO (orange squares) and GPSG-BO (red triangles) compared to the previous parameterization (derived using optimization with a genetic algorithm, GA-O, black crosses). Grey dots show the calibration data.

6 VALIDATION

A

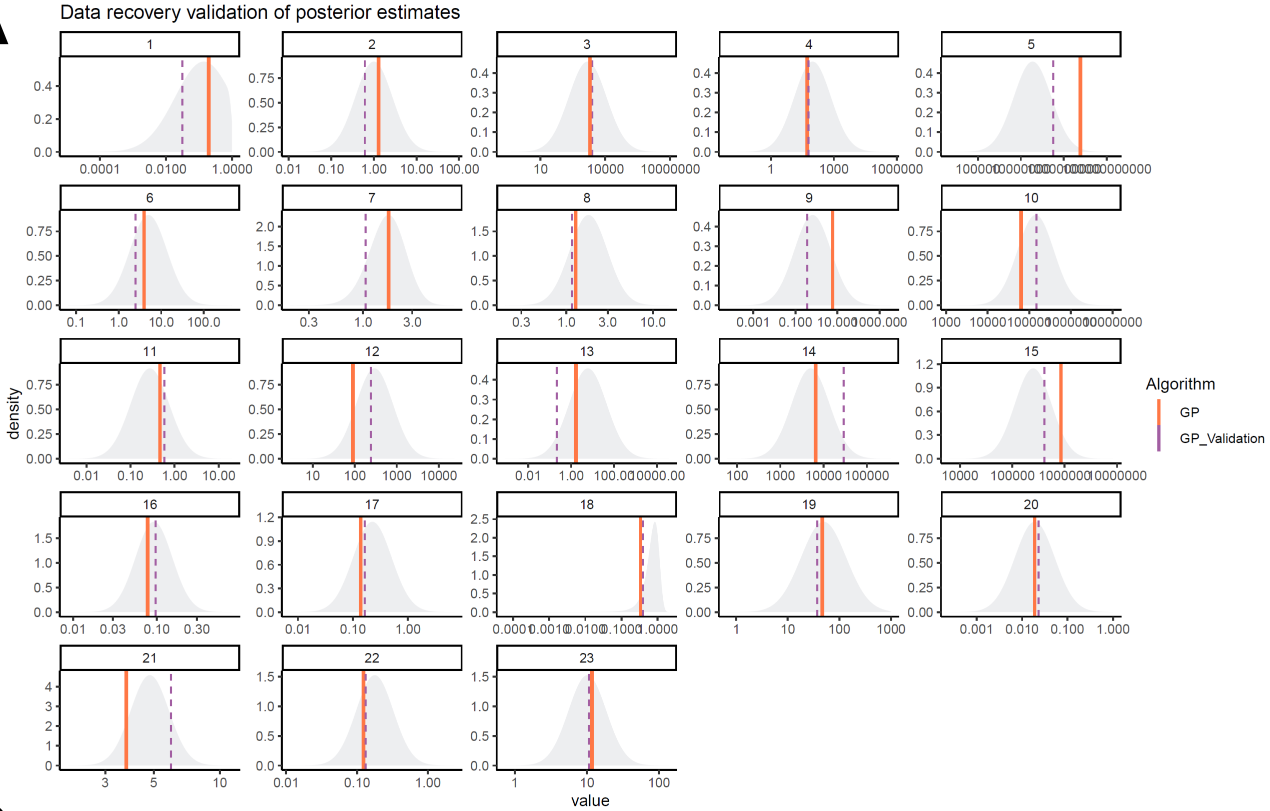

B

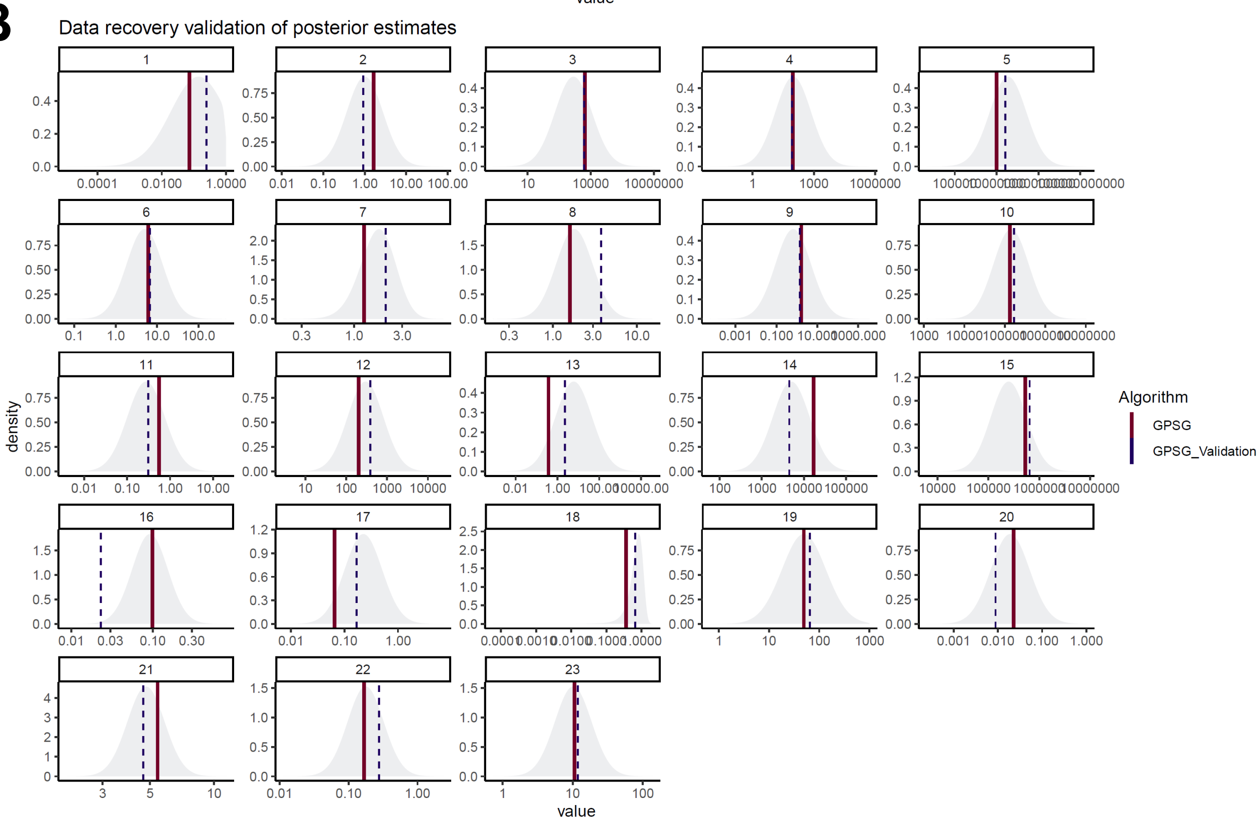

Supplementary Figure 19. Data recovery validation of posterior estimates. Prior distributions of each of the 23 parameters and parameter value identified by the optimization algorithm. The final parameter set was used to generate synthetic field data by simulating each of the 61 scenarios with the respective core parameter sets. Simulation outputs were reformatted to match the original field data, generating a synthetic field data set. The optimization with both algorithms was repeated using this synthetic field data. The plots show the best parameter values in each dimension identified at the end of the validation optimization (dashed lines) compared with the values identified in the original optimization (solid lines). The grey areas show the prior distribution. A. GP-BO validation. B. GPSG-BO validation.

7 OPENMALARIA SIMULATED EPIDEMIOLOGY

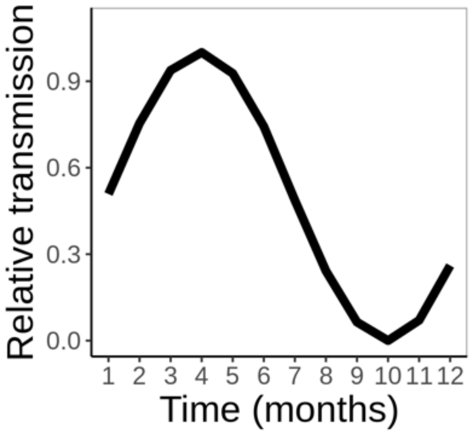

Supplementary Figure 20. Seasonal pattern assumed for subsequent analyses. The monthly transmission intensity is equivalent to the transmission intensity relative to the annual total (entomological inoculation rate, EIR) scaled by these values and forced to sum to the annual EIR.

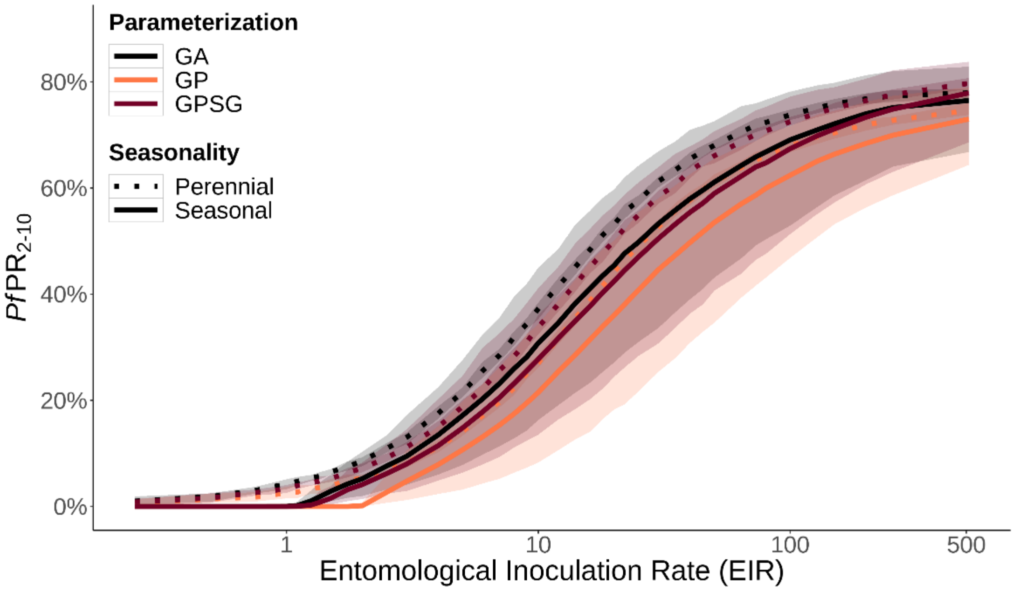

Supplementary Figure 21. Relationship between EIR and *Pf*PR$_{2\text{-}10}$ under three parameterizations. Solid lines show medians and shaded areas show the minimum to maximum range. EIR denotes the entomological inoculation rate.

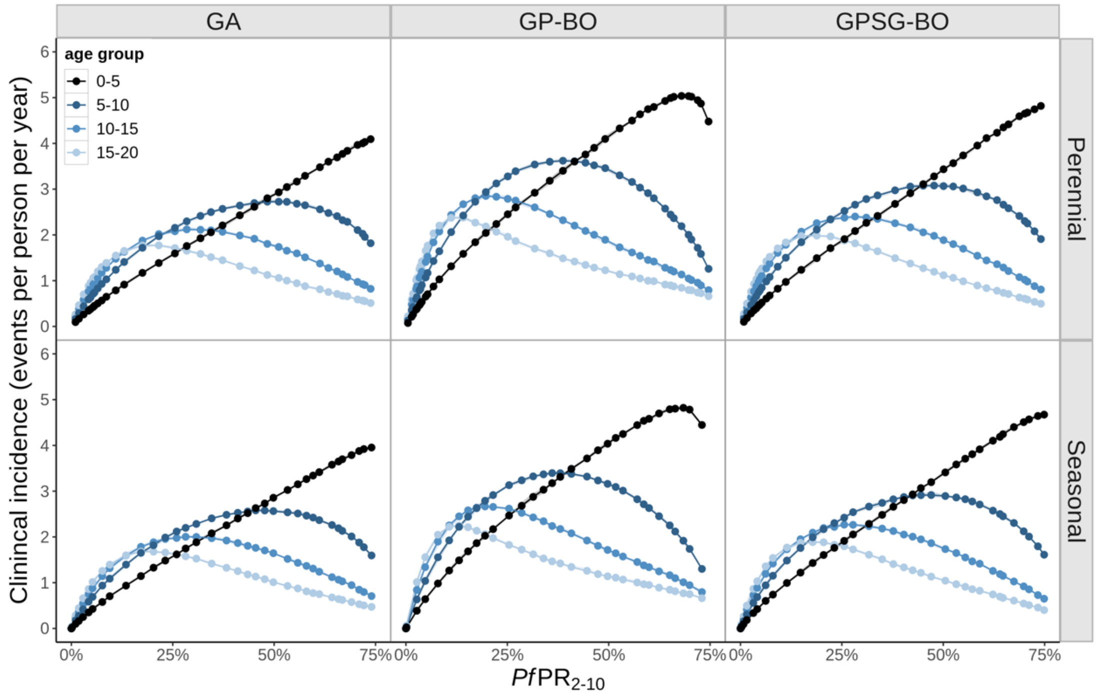

Supplementary Figure 22. Yearly incidence of clinical (uncomplicated) malaria as a function of *Pf*PR$_{2-10}$ displayed by parameterization and age group (in years). Clinical incidence is presented in terms of the yearly number of events per person. The shaded areas show the minimum to maximum range. We assume a probability of effective treatment within 14 days of uncomplicated malaria of 36%.

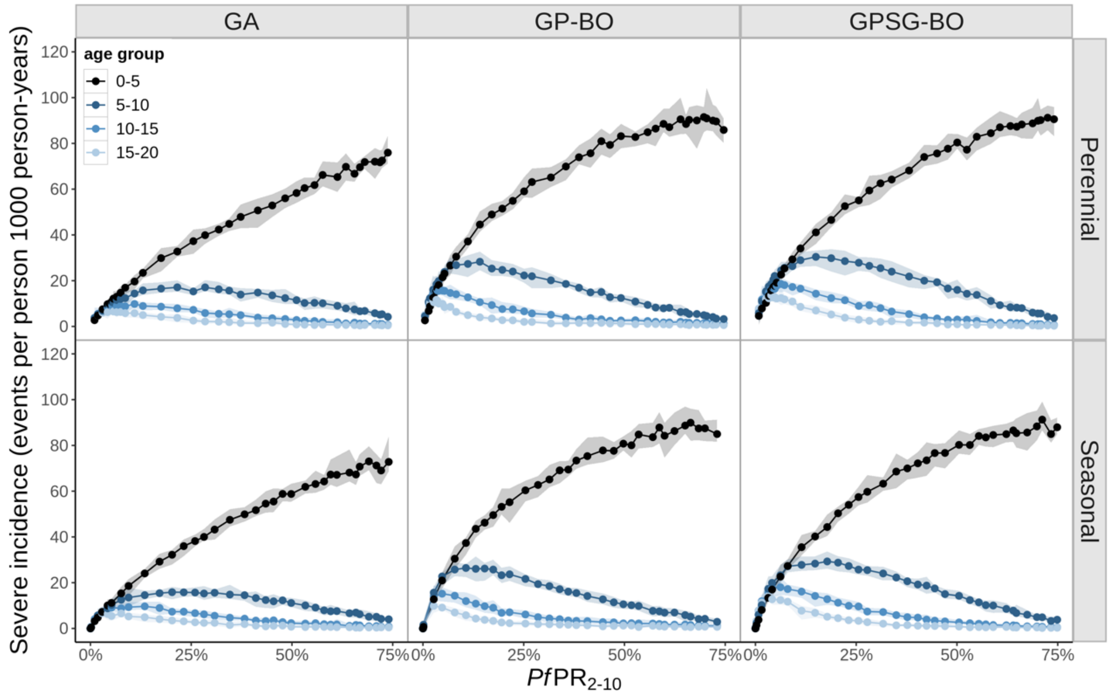

Supplementary Figure 23. Yearly incidence of total severe malaria as a function of *Pf*PR$_{2-10}$, displayed by parameterization and age group (in years). Incidence is presented in terms of the yearly number of events in a population of 1000 individuals. The shaded areas show the minimum to maximum range. It is assumed that 48% of severe malaria cases seek official care at a heath care facility (hospital). We assume a probability of effective treatment within 14 days of uncomplicated malaria of 36%.

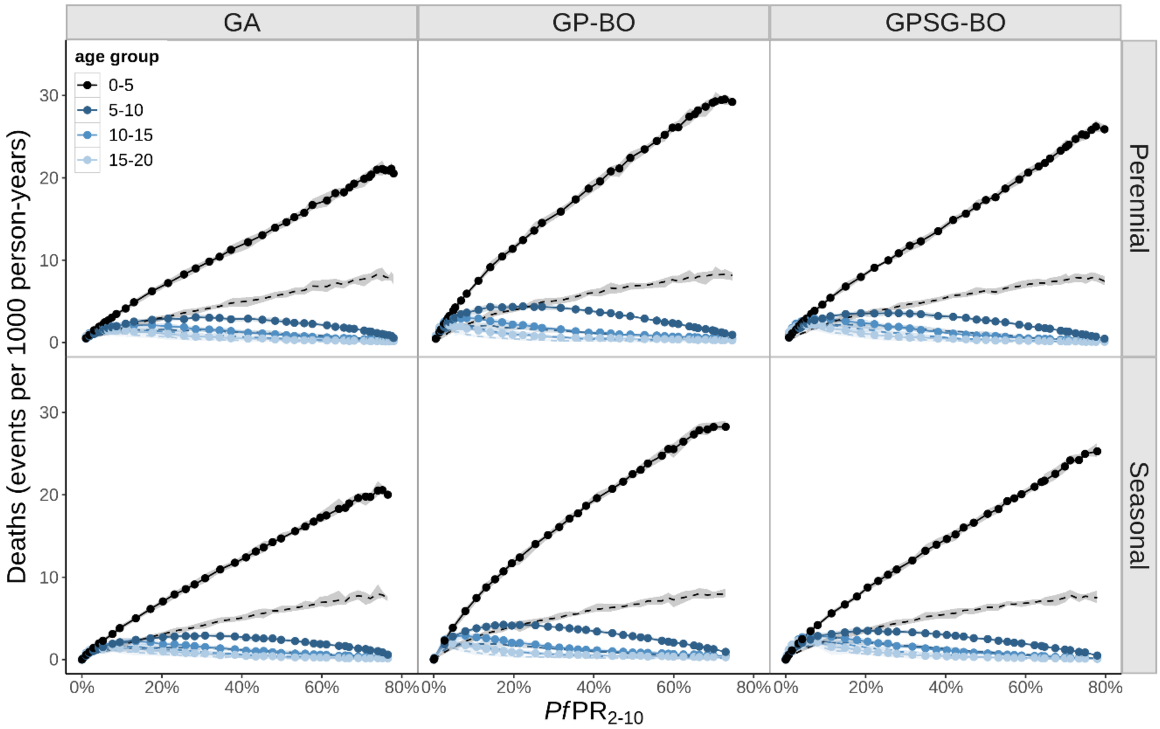

Supplementary Figure 24. Yearly number of malaria-related deaths as a function of *Pf*PR$_{2\text{-}10}$, displayed by parameterization and age group (in years). Malaria mortality incidence is presented in terms of the yearly number of deaths in a population of 1000 individuals. For the OpenMalaria model both deaths directly attributed to malaria (dotted curve) and all deaths associated with malaria (including both deaths directly attributable to malaria and those associated with comorbidities) are shown (full line). The shaded areas show the minimum to maximum range.

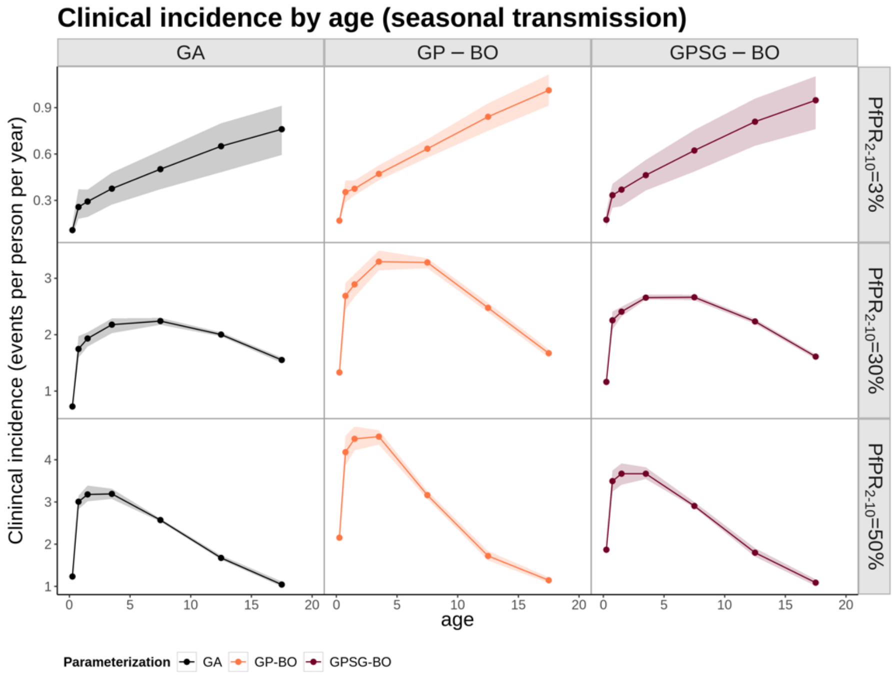

Supplementary Figure 25. Yearly incidence of clinical malaria in a seasonal transmission setting as a function of age, displayed by transmission intensity (*Pf*PR$_{2-10}$) and parameterization. Clinical incidence is presented in terms of the yearly number of events per person. The *Pf*PR$_{2-10}$ categories include simulated prevalences of 2.5-3.5%, 9-10%, 28-32%, and 47-53% labeled as 3%, 10%, 30%, and 50%, respectively. The shaded areas show the minimum to maximum range.

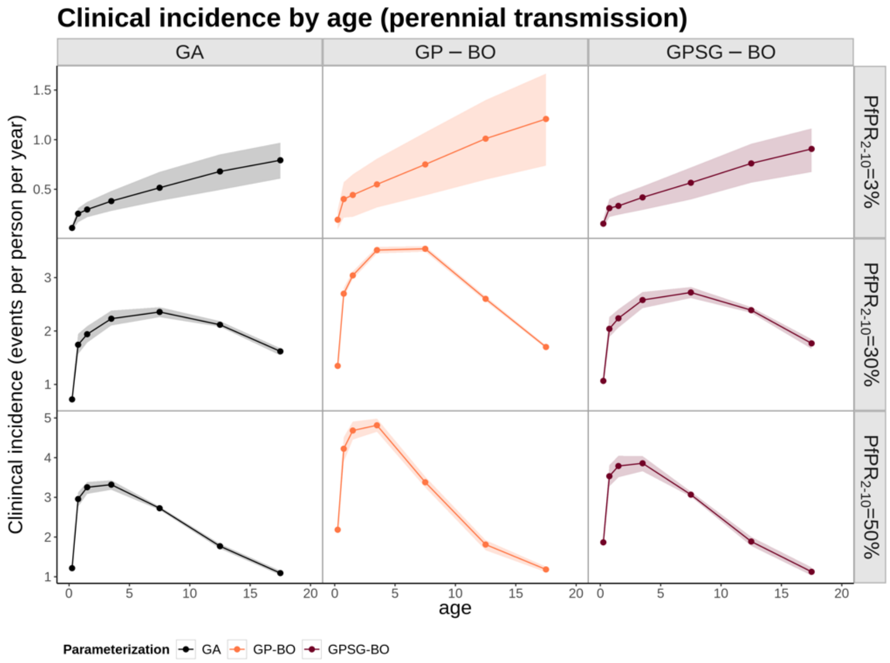

Supplementary Figure 26. Yearly incidence of clinical malaria in a perennial transmission setting as a function of age (in years), displayed by transmission intensity (*Pf*PR$_{2-10}$), and parameterization. Clinical incidence is presented in terms of the yearly number of events per person. The *Pf*PR$_{2-10}$ categories include simulated prevalences of 2.5-3.5%, 9-10%, 28-32%, and 47-53% labeled as 3%, 10%, 30%, and 50%, respectively. The shaded areas show the minimum to maximum range.

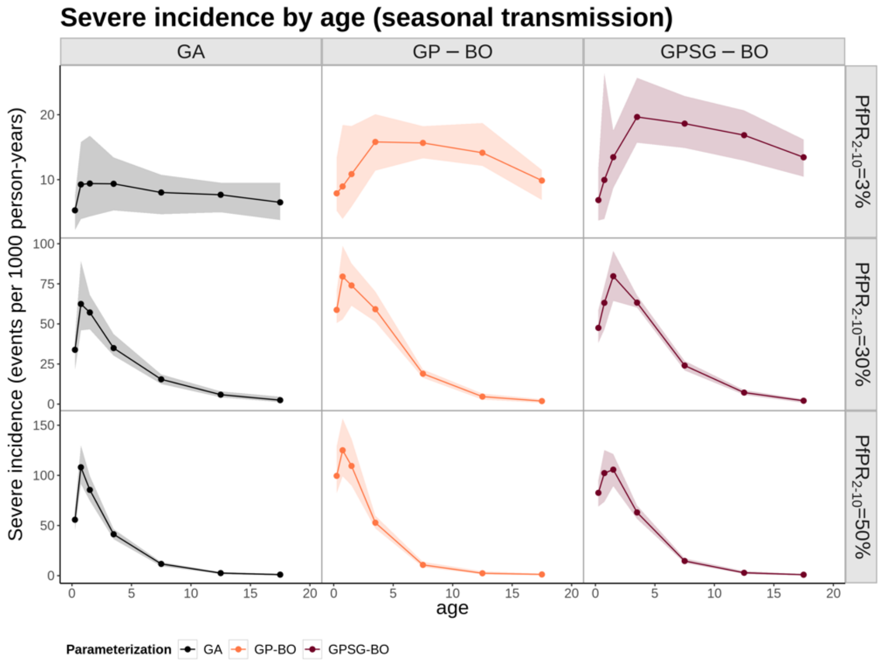

Supplementary Figure 27. Yearly incidence of total severe malaria in a seasonal transmission setting as a function of age (in years), displayed by transmission intensity (*Pf*PR$_{2-10}$), and parameterization. Incidence is presented in terms of the yearly number of events per 1000 person-years. It is assumed that 48% of severe malaria cases seek official care at a healthcare facility (hospital). The *Pf*PR$_{2-10}$ categories include simulated prevalences of 2.5-3.5%, 9-10%, 28-32%, and 47-53% labeled as 3%, 10%, 30%, and 50%, respectively. The shaded areas show the minimum to maximum range.

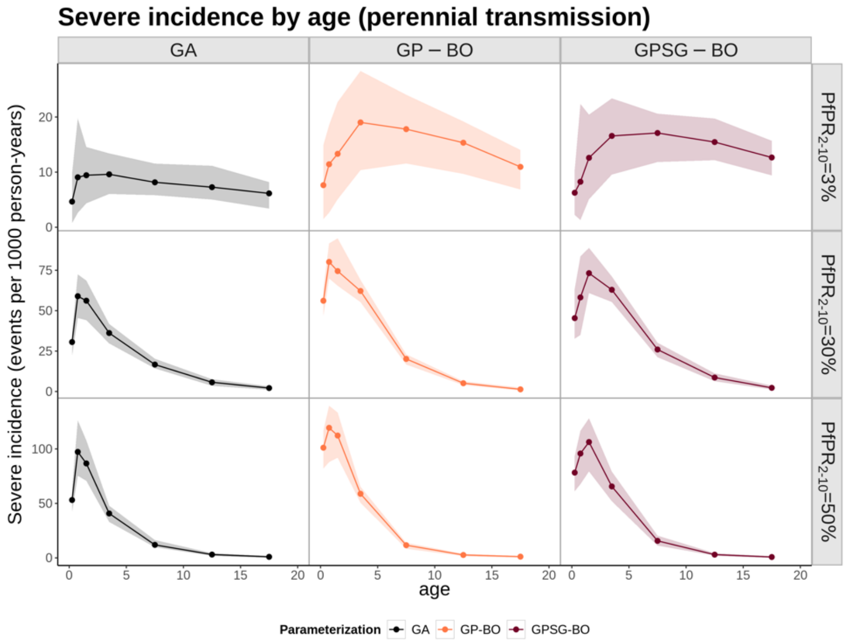

Supplementary Figure 28. Yearly incidence of total severe malaria in a perennial transmission setting as a function of age (in years), displayed by transmission intensity (*Pf*PR$_{2-10}$), and parameterization. Incidence is presented in terms of the yearly number of events per 1000 person-years. It is assumed that 48% of severe malaria cases seek official care at a healthcare facility (hospital). The *Pf*PR$_{2-10}$ categories include simulated prevalences of 2.5-3.5%, 9-10%, 28-32%, and 47-53% labeled as 3%, 10%, 30%, and 50%, respectively. The shaded areas show the minimum to maximum range.

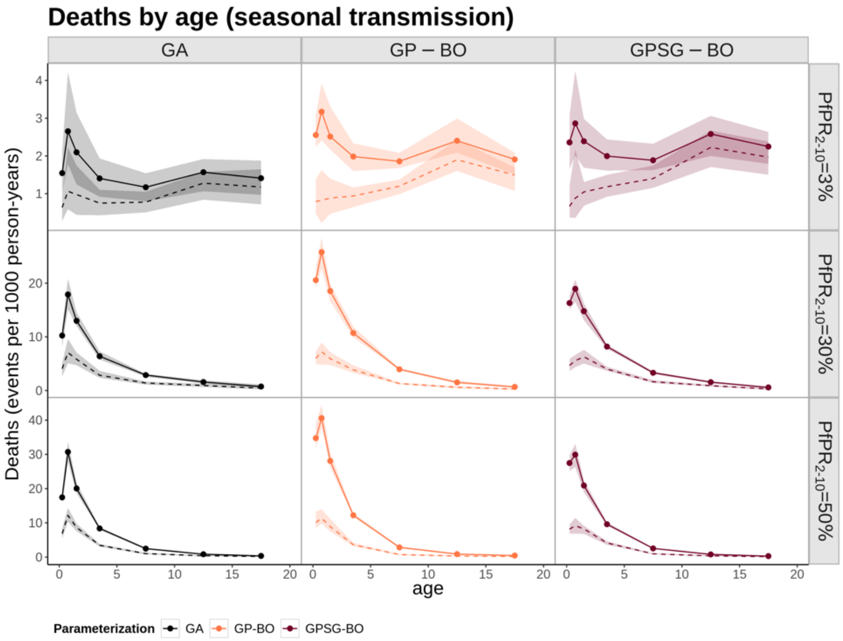

Supplementary Figure 29. Yearly incidence of malaria-related deaths in a seasonal transmission setting as a function of age, displayed by transmission intensity (*Pf*PR$_{2\text{-}10}$) and parameterization. Malaria mortality incidence is presented in terms of the yearly number of deaths in a population of 1000 individuals. The dashed estimates represent direct malaria deaths, and the solid, all malaria deaths (including those attributable to co-morbidities). The shaded areas show the minimum to maximum range. The *Pf*PR$_{2\text{-}10}$ categories include simulated prevalences of 2.5-3.5%, 9-10%, 28-32%, and 47-53% labeled as 3%, 10%, 30%, and 50%, respectively.

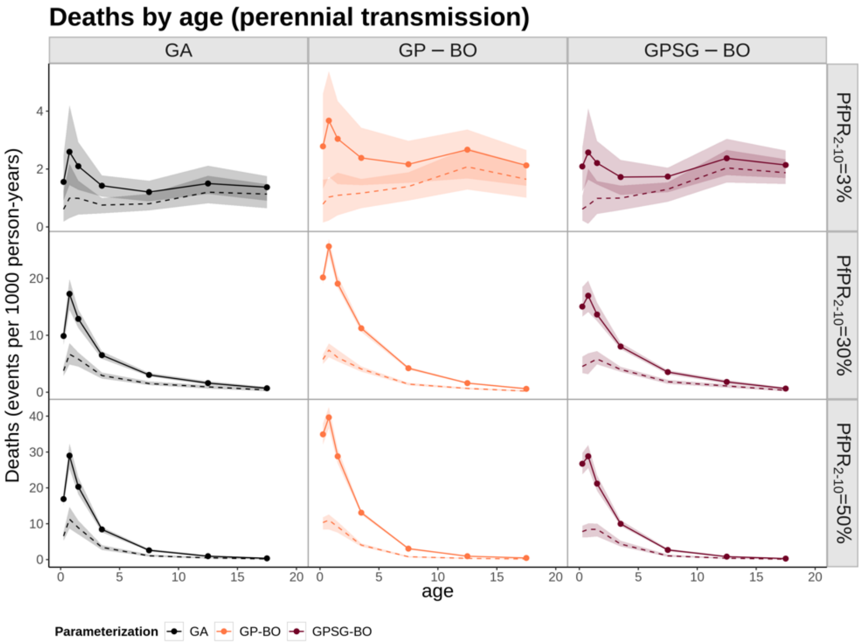

Supplementary Figure 30. Yearly incidence of malaria-related deaths in a perennial transmission setting as a function of age, displayed by transmission intensity (*Pf*PR$_{2\text{-}10}$) and parameterization. Malaria mortality incidence is presented in terms of the yearly number of deaths in a population of 1000 individuals. The dashed estimates represent direct malaria deaths, and the solid, all malaria deaths (including those attributable to co-morbidities). The shaded areas show the minimum to

maximum range. The *Pf*PR$_{2-10}$ categories include simulated prevalences of 2.5-3.5%, 9-10%, 28-32%, and 47-53% labeled as 3%, 10%, 30%, and 50%, respectively.

8 LOG PRIOR DISTRIBUTIONS

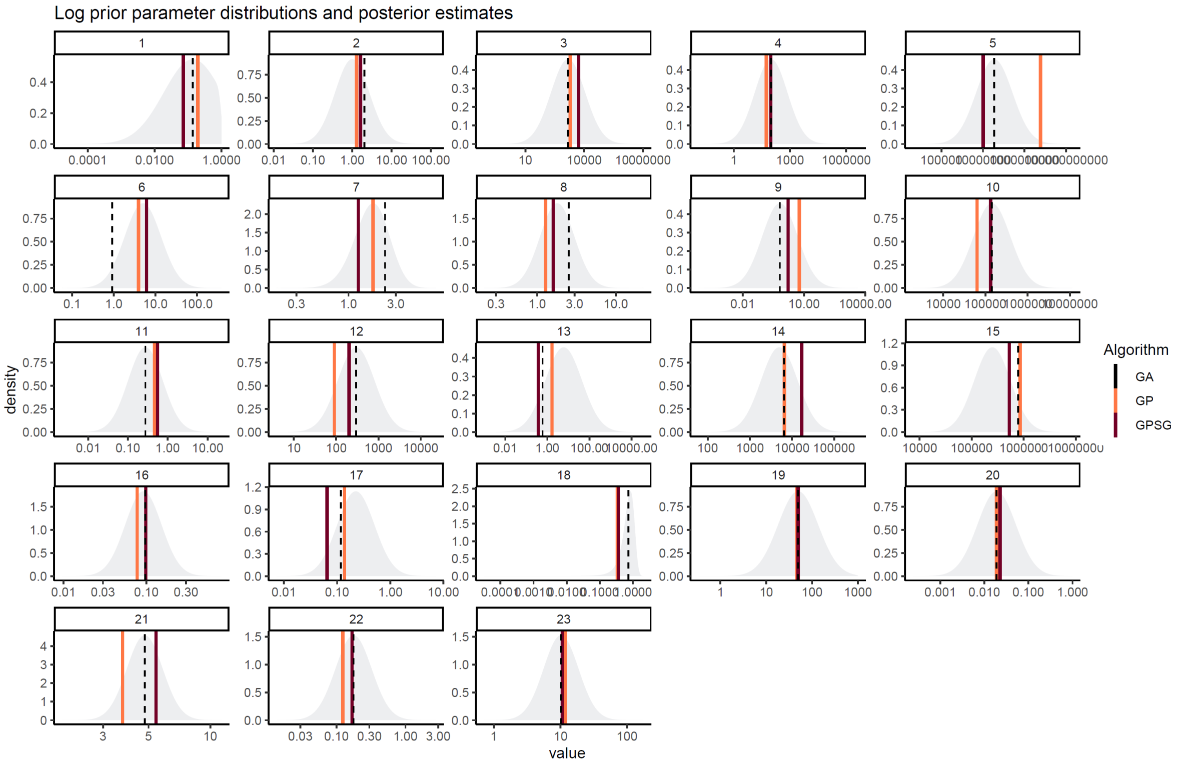

Supplementary Figure 31. Log prior distributions and final posterior estimates. Prior distributions of each parameter (grey areas) and final parameter values identified by each optimization algorithm (GP-BO and GPSG-BO) and compared to the current parameterization (derived using a genetic algorithm, GA).

9 RANGER IMPORTANCE

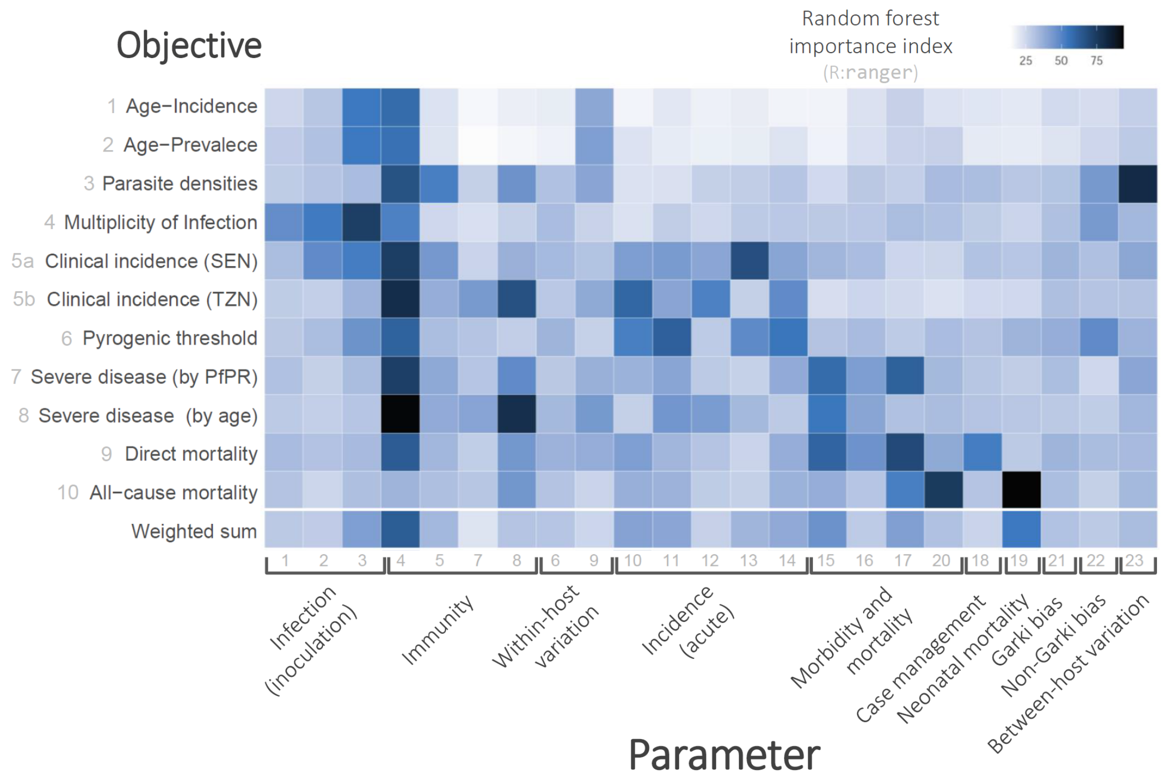

Supplementary Figure 32. Random forest importance. Estimated parameter importance indices for all parameters and objectives. The indices were calculated using the ranger random forest package in R.

10 REFERENCES

1 Smith, T. *et al.* Ensemble modeling of the likely public health impact of a pre-erythrocytic malaria vaccine. *PLoS Med* **9**, e1001157 (2012).

2 Smith, T. *et al.* Mathematical modeling of the impact of malaria vaccines on the clinical epidemiology and natural history of Plasmodium falciparum malaria: Overview. *The American Journal of Tropical Medicine and Hygiene* **75**, 1-10 (2006).

3 Maire, N. *et al.* A model for natural immunity to asexual blood stages of Plasmodium falciparum malaria in endemic areas. *The American Journal of Tropical Medicine and Hygiene* **75**, 19-31 (2006).

4 Collins, W. E. & Jeffery, G. M. A retrospective examination of sporozoite-and trophozoite-induced infections with Plasmodium falciparum: development of parasitologic and clinical immunity during primary infection. *The American Journal of Tropical Medicine and Hygiene* **61**, 4-19 (1999).

5 Smith, T. *et al.* Relationship between the entomologic inoculation rate and the force of infection for Plasmodium falciparum malaria. *The American Journal of Tropical Medicine and Hygiene* **75**, 11-18 (2006).

6 Killeen, G. F., Ross, A. & Smith, T. Infectiousness of malaria-endemic human populations to vectors. *The American Journal of Tropical Medicine and Hygiene* **75**, 38-45 (2006).

7 Ross, A., Killeen, G. & Smith, T. Relationships between host infectivity to mosquitoes and asexual parasite density in Plasmodium falciparum. *The American Journal of Tropical Medicine and Hygiene* **75**, 32-37 (2006).

8 Carneiro, I. A. *et al.* Modeling the relationship between the population prevalence of Plasmodium falciparum malaria and anemia. *The American Journal of Tropical Medicine and Hygiene* **75**, 82-89 (2006).

9 Ross, A. & Smith, T. The effect of malaria transmission intensity on neonatal mortality in endemic areas. *The American Journal of Tropical Medicine and Hygiene* **75**, 74-81 (2006).

10 Smith, T. *et al.* An epidemiologic model of the incidence of acute illness in Plasmodium falciparum malaria. *The American Journal of Tropical Medicine and Hygiene* **75**, 56-62 (2006).

11 Ross, A., Maire, N., Molineaux, L. & Smith, T. An epidemiologic model of severe morbidity and mortality caused by Plasmodium falciparum. *The American Journal of Tropical Medicine and Hygiene* **75**, 63-73 (2006).

12 Port, G., Boreham, P. & Bryan, J. H. The relationship of host size to feeding by mosquitoes of the Anopheles gambiae Giles complex (Diptera: Culicidae). *Bulletin of Entomological Research* **70**, 133-144 (1980).

13 H., R. *et al.* The epidemiology of severe malaria due to *Plasmodium falciparum* at different transmisison intensities in NE Tanzania. (2004).

14 Molineaux, L., Gramiccia, G. & Organization, W. H. *The Garki project: research on the epidemiology and control of malaria in the Sudan savanna of West Africa*. (World Health Organization, 1980).

15 Owusu-Agyei, S., Smith, T., Beck, H. P., Amenga-Etego, L. & Felger, I. Molecular epidemiology of Plasmodium falciparum infections among asymptomatic inhabitants of a holoendemic malarious area in northern Ghana. *Tropical Medicine & International Health* **7**, 421-428 (2002).

16 Trape, J.-F. & Rogier, C. Combating malaria morbidity and mortality by reducing transmission. *Parasitology today* **12**, 236-240 (1996).

17 Kitua, A. Y. *et al.* Plasmodium falciparum malaria in the first year of life in an area of intense and perennial transmission. *Tropical Medicine & International Health* **1**, 475-484 (1996).

18 Rogier, C., Commenges, D. & Trape, J.-F. Evidence for an age-dependent pyrogenic threshold of Plasmodium falciparum parasitemia in highly endemic populations. *The American Journal of Tropical Medicine and Hygiene* **54**, 613-619 (1996).

19 Marsh, K. & Snow, R. Malaria transmission and morbidity. *Parassitologia* **41**, 241 (1999).

20 Snow, R. W. *et al.* Relation between severe malaria morbidity in children and level of Plasmodium falciparum transmission in Africa. *The Lancet* **349**, 1650-1654 (1997).

21 Smith, T. *et al.* Absence of seasonal variation in malaria parasitaemia in an area of intense seasonal transmission. *Acta Tropica* **54**, 55-72 (1993).

22 Earle, W. C. & Perez, M. Enumeration of parasites in the blood of malarial patients. *Journal of Laboratory and Clinical Medicine* **17** (1932).

23 Trape, J.-F. *et al.* The Dielmo project: a longitudinal study of natural malaria infection and the mechanisms of protective immunity in a community living in a holoendemic area of Senegal. *The American Journal of Tropical Medicine and Hygiene* **51**, 123-137 (1994).

24 Charlwood, J. *et al.* Incidence of Plasmodium falciparum infection in infants in relation to exposure to sporozoite-infected anophelines. *The American Journal of Tropical Medicine and Hygiene* **59**, 243-251 (1998).

25 Korenromp, E. L., Williams, B. G., Gouws, E., Dye, C. & Snow, R. W. Measurement of trends in childhood malaria mortality in Africa: an assessment of progress toward targets based on verbal autopsy. *The Lancet Infectious Diseases* **3**, 349-358 (2003).

26 Vounatsou, P., Smith, T., Kitua, A., Alonso, P. & Tanner, M. Apparent tolerance of Plasmodium falciparum in infants in a highly endemic area. *Parasitology* **120**, 1-9 (2000).

27 Beier, J. C., Killeen, G. F. & Githure, J. I. Entomologic inoculation rates and Plasmodium falciparum malaria prevalence in Africa. *The American Journal of Tropical Medicine and Hygiene* **61**, 109-113 (1999).

28 Barnish, G. *et al.* Malaria in a rural area of Sierra Leone. I. Initial results. *Annals of Tropical Medicine & Parasitology* **87**, 125-136 (1993).

29 Centre, I. D. R. & Network, I. *Population and Health in Developing Countries: Population, health and survival at INDEPTH sites*. Vol. 1 (IDRC, 2002).

30 Spencer, H. C. *et al.* Impact on mortality and fertility of a community-based malaria control programme in Saradidi, Kenya. *Annals of Tropical Medicine & Parasitology* **81**, 36-45 (1987).

31 D'Alessandro, U. *et al.* Mortality and morbidity from malaria in Gambian children after introduction of an impregnated bednet programme. *The Lancet* **345**, 479-483 (1995).

32 Duboz, P., Vaugelade, J. & Debouverie, M. Mortalité dans l'enfance dans la région de Niangoloko. (ORSTOM, Ouagadougou, Burkina Faso, 1989).

33 Schellenberg, J. A. *et al.* KINET: a social marketing programme of treated nets and net treatment for malaria control in Tanzania, with evaluation of child health and long-term survival. *Transactions of the Royal Society of Tropical Medicine and Hygiene* **93**, 225-231 (1999).

34 Premji, Z. *et al.* Community based studies on childhood mortality in a malaria holoendemic area on the Tanzanian coast. *Acta Tropica* **63**, 101-109 (1997).

35 Trape, J.-F. *et al.* Impact of chloroquine resistance on malaria mortality. *Comptes Rendus de l'Académie des Sciences-Series III-Sciences de la Vie* **321**, 689-697 (1998).
