# Supplementary material for: Emulator-based Bayesian optimization for efficient multi-objective calibration of an individual-based model of malaria
Source: Nat Commun. 2021 Dec 10;12:7212. doi: 10.1038/s41467-021-27486-z (PMC8664949; doi:10.1038/s41467-021-27486-z)
Supplement: Supplementary file 3 — Reporting Summary [file 41467_2021_27486_MOESM3_ESM.pdf]

nature portfolio

Corresponding author(s): Melissa Penny
Last updated by author(s): Oct 31, 2021

Reporting Summary

Nature Portfolio wishes to improve the reproducibility of the work that we publish. This form provides structure for consistency and transparency in reporting. For further information on Nature Portfolio policies, see our Editorial Policies and the Editorial Policy Checklist.

Statistics

For all statistical analyses, confirm that the following items are present in the figure legend, table legend, main text, or Methods section.

n/a	Confirmed	
☐	☒	The exact sample size (*n*) for each experimental group/condition, given as a discrete number and unit of measurement
☒	☐	A statement on whether measurements were taken from distinct samples or whether the same sample was measured repeatedly
☒	☐	The statistical test(s) used AND whether they are one- or two-sided<br>*Only common tests should be described solely by name; describe more complex techniques in the Methods section.*
☒	☐	A description of all covariates tested
☒	☐	A description of any assumptions or corrections, such as tests of normality and adjustment for multiple comparisons
☐	☒	A full description of the statistical parameters including central tendency (e.g. means) or other basic estimates (e.g. regression coefficient) AND variation (e.g. standard deviation) or associated estimates of uncertainty (e.g. confidence intervals)
☒	☐	For null hypothesis testing, the test statistic (e.g. *F*, *t*, *r*) with confidence intervals, effect sizes, degrees of freedom and *P* value noted<br>*Give P values as exact values whenever suitable.*
☐	☒	For Bayesian analysis, information on the choice of priors and Markov chain Monte Carlo settings
☐	☒	For hierarchical and complex designs, identification of the appropriate level for tests and full reporting of outcomes
☒	☐	Estimates of effect sizes (e.g. Cohen's *d*, Pearson's *r*), indicating how they were calculated

Our web collection on statistics for biologists contains articles on many of the points above.

Software and code

Policy information about availability of computer code

Data collection	To inform the estimation of core parameters, epidemiological data on the natural history of malaria were extracted from published literature and collated in previous calibrations of OpenMalaria (3, 21, 24). These include demographic data such as age-stratified numbers of host individuals. Data sources and descriptions by objective are detailed in the Supplement Text 2.
Data analysis	Consistent with previous calibration work, we used OpenMalaria version 35, an open-source simulator written in C++ and further detailed in full in the supplement, in the OpenMalaria wiki (https://github.com/SwissTPH/openmalaria/wiki), or in the original publications (3, 21, 24). Calibration was performed using R 3.6.0. Previously the model was calibrated using an asynchronous genetic algorithm (3, 21, 24). For the machine learning processes, all algorithms were accessed through the mlr package in R version 2.17.0 (66). The heteroskedastic Gaussian process (GP) utilised the hetGP package under version 1.1.2 (65). To emulate the solution space, the GP emulator (39) and a superlearning algorithm in form of a Gaussian process stacked generalization (GPSG) emulator (40) were compared. In superlearning, the cross-validated predictions of the level 0 learners are fed into a level 1 meta-learner. We compared the 10-fold cross-validated predictive performance of twelve machine learning algorithms on the test set. All algorithms were accessed through the mlr package in R version 2.17.0 (66). We compared two neural network algorithms (brnn (54) for a two-layer neural network and nnet for a single-hidden-layer neural network (67)), five regression algorithms (cvglmnet (68) for a generalised linear model with LASSO or Elasticnet Regularization and 10-fold cross validated lambda, glmboost (69) for a boosted generalized linear model, glmnet (68) for a regular GLM with Lasso or Elasticnet Regularisation, mars for multivariate adaptive regression splines (70), and cubist for rule-and instance-based regression modelling (71)), three random forest algorithms (randomForest (58), randomForestSRC (72) and ranger (73)), and a tree-like node harvesting algorithm (nodeHarvest (74)). The sensitivity analysis was conducted using the soboljansen function of the sensitivity package version 1.21.0 in R (77). All algorithms were adapted to the operating system (CentOS 7.5.1804) and computational resources available at the University of Basel Center for Scientific Computing, SciCORE, which uses a Slurm queueing system were used. The full algorithm code is available on GitHub (https://github.com/reikth/BayesOpt_Calibration).

For manuscripts utilizing custom algorithms or software that are central to the research but not yet described in published literature, software must be made available to editors and reviewers. We strongly encourage code deposition in a community repository (e.g. GitHub). See the Nature Portfolio guidelines for submitting code & software for further information.

Data

Policy information about availability of data

All manuscripts must include a data availability statement. This statement should provide the following information, where applicable:

- Accession codes, unique identifiers, or web links for publicly available datasets
- A description of any restrictions on data availability
- For clinical datasets or third party data, please ensure that the statement adheres to our policy

All calibration data are detailed in (24) and further available from the researchers on request.

Field-specific reporting

Please select the one below that is the best fit for your research. If you are not sure, read the appropriate sections before making your selection.

☒ Life sciences ☐ Behavioural & social sciences ☐ Ecological, evolutionary & environmental sciences

For a reference copy of the document with all sections, see nature.com/documents/nr-reporting-summary-flat.pdf

Life sciences study design

All studies must disclose on these points even when the disclosure is negative.

Sample size	To compare the goodness of fit for the age-specific multiplicity of infection, simulations were carried out for a population size of N=5,000 (as shown in figure 3A). To simulate the epidemiological relationship between the transmission intensity (entomological inoculation rate, EIR) and infection prevalence in individuals aged 2-10 years (PfPR2-10) under the parameterizations achieved by the different optimization algorithms, a simulated population size of N=10,000 was applied (as shown in figure 3B). A global sensitivity analysis was conducted on a heteroskedastic GP model that was trained on all training simulation outputs (n=5,400) from the fitting process (as shown in figure 3C).
Data exclusions	No data were excluded from the analyses.
Replication	Not applicable for data use. For algorithms developed to calibrate models, we tested them on synthetic data to test we could recover the parameter values used to create the synthetic data.
Randomization	Randomization was not applicable.
Blinding	Blinding was not applicable.

Reporting for specific materials, systems and methods

We require information from authors about some types of materials, experimental systems and methods used in many studies. Here, indicate whether each material, system or method listed is relevant to your study. If you are not sure if a list item applies to your research, read the appropriate section before selecting a response.

Materials & experimental systems

n/a	Involved in the study	
☒	☐	Antibodies
☒	☐	Eukaryotic cell lines
☒	☐	Palaeontology and archaeology
☒	☐	Animals and other organisms
☒	☐	Human research participants
☒	☐	Clinical data
☒	☐	Dual use research of concern

Methods

n/a	Involved in the study	
☒	☐	ChIP-seq
☒	☐	Flow cytometry
☒	☐	MRI-based neuroimaging
